# Electrophysiological properties of human beta-cell lines EndoC-βH1 and -βH2 conform with human beta-cells

**DOI:** 10.1038/s41598-018-34743-7

**Published:** 2018-11-19

**Authors:** Benoît Hastoy, Mahdieh Godazgar, Anne Clark, Vibe Nylander, Ioannis Spiliotis, Martijn van de Bunt, Margarita V. Chibalina, Amy Barrett, Carla Burrows, Andrei I. Tarasov, Raphael Scharfmann, Anna L. Gloyn, Patrik Rorsman

**Affiliations:** 1 Oxford Centre for Diabetes, Endocrinology and Metabolism (OCDEM), Radcliffe Department of Medicine, University of Oxford, Oxford, United Kingdom; 2 Wellcome Centre for Human Genetics, Nuffield Department of Medicine, University of Oxford, Oxford, United Kingdom; 3 INSERM U1016, Cochin Institute, Université Paris Descartes, Paris, France; 4 National Institute for Health Research (NIHR) Oxford Biomedical Research Centre, Churchill Hospital, Oxford, United Kingdom; 5 Department of Physiology, Institute of Neuroscience and Physiology, University of Goteborg, Goteborg, Sweden

## Abstract

Limited access to human islets has prompted the development of human beta cell models. The human beta cell lines EndoC-βH1 and EndoC-βH2 are increasingly used by the research community. However, little is known of their electrophysiological and secretory properties. Here, we monitored parameters that constitute the glucose-triggering pathway of insulin release. Both cell lines respond to glucose (6 and 20 mM) with 2- to 3-fold stimulation of insulin secretion which correlated with an elevation of [Ca^2+^]_i_, membrane depolarisation and increased action potential firing. Similar to human primary beta cells, K_ATP_ channel activity is low at 1 mM glucose and is further reduced upon increasing glucose concentration; an effect that was mimicked by the K_ATP_ channel blocker tolbutamide. The upstroke of the action potentials reflects the activation of Ca^2+^ channels with some small contribution of TTX-sensitive Na^+^ channels. The repolarisation involves activation of voltage-gated Kv2.2 channels and large-conductance Ca^2+^-activated K^+^ channels. Exocytosis presented a similar kinetics to human primary beta cells. The ultrastructure of these cells shows insulin vesicles composed of an electron-dense core surrounded by a thin clear halo. We conclude that the EndoC-βH1 and -βH2 cells share many features of primary human β-cells and thus represent a useful experimental model.

## Introduction

Electrical activity plays a critical role in glucose-stimulated insulin secretion (GSIS)^1,2^. An understanding of the stimulus-secretion coupling in beta-cells is important as its dysfunction is recognised to be a central feature of Type 2 Diabetes (T2D)^3,4^. Indeed, the majority of genome-wide association study (GWAS) loci identified to date for T2D affect beta-cell function and/or mass^5,6^. However, exactly how these variants impact beta-cell function has only been established for a handful of them.

The limited availability of human islets preparations coupled with donor variability has hampered the study of human beta-cell function. Consequently, determining how genetic variants and the transcripts they exert their effect on influence beta-cell function remains a challenging topic to explore. Therefore, access to a human beta-cell line amenable to genetic modification would be extremely valuable. The EndoC-βH1 and -βH2 cells were generated from human foetal pancreatic buds and express numerous beta-cell markers. These human beta-cell lines respond to elevated glucose with stimulation of insulin secretion^7,8^ and are increasingly used to explore various aspects of human beta-cell biology^9–21^.

Here, we monitored different parameters that constitute the triggering pathway of GSIS^1,22^ and the electrophysiological and ultrastructural properties of EndoC-βH1 and -βH2 cells. We correlate our electrophysiological characterisation with global gene transcript levels for both cell lines. Overall, our data show consistency between the EndoC-βH1 and -βH2 cells and primary human beta-cells, supporting their use as a valuable model system.

## Methods

### Ethics

Human pancreatic islets were isolated from deceased donors under ethical approval obtained from the human research ethics committees in Oxford (REC: 09/H0605/2, NRES committee South Central-Oxford B). All donors gave informed research consent as part of the national organ donation programme. Islets were obtained from the Diabetes Research & Wellness Foundation Human Islet Isolation Facility, OCDEM, University of Oxford. All methods and protocols using human pancreatic islets were performed in accordance with the relevant guidelines and regulations in the UK (Human Tissue Authority, HTA).

### Cell lines and cell culture

EndoC-βH1 and -βH2 cell lines, both generated from human fetal pancreatic buds were provided by Endocell and Raphael Scharfmann^7,8^. Both cell lines were regularly tested for mycoplasma contamination and cultured as previously published^8^. Additional details are available in the Supplementary material.

### Insulin Secretion

EndoC-βH1 and βH2 cells were seeded onto coated 24 well plates at a density of 300,000 cells/well. The night before experiment, the cells were incubated in 2.8 mmol/L glucose culture medium. Prior to the experiment, the cells were incubated in a modified Krebs-Ringer buffer (KRB) medium consisting of (mmol/L) 138 NaCl, 3.6 KCl, 0.5 MgSO_4_, 0.5 NaH_2_PO_4_, 5 NaHC0_3_, 1.5 CaCl_2_ and 5 HEPES (adjusted to pH 7.4 with NaOH) and supplemented with 0.2% w/v BSA. The cells were washed with the glucose-free medium, preincubated for 15 min at 1 mmol/L glucose before a 40 min test incubation at either 1, 6 or 20 mmol/L glucose and with added tolbutamide (0.2 mmol/L) or diazoxide (0.5 mmol/L) as indicated. Supernatants (0.3 ml) were taken for determination of insulin release. Cellular insulin content was extracted by acid ethanol treatment. The samples were frozen pending later analysis which was carried out using commercial ELISA (Alpha Laboratories) or radioimmunosassay (HI-14 K, Merck). Insulin secretion is expressed as a percentage of cellular insulin content and fold-increase in secretion as a ratio with basal secretion (1 mmol/L). The somatostatin receptor 2 (SSTR2) antagonist CYN154806 was obtained from TOCRIS (Abingdon, UK).

### Electrophysiology

All electrophysiological experiments were performed as described previously^23^. Agatoxin, isradipine, SNX 482, stromatoxin and iberiotoxin were purchased from Alomone (Jerusalem, Israel) and tetrodotoxin (TTX) from Sigma (Gillingham, UK).

#### Solutions

All electrophysiological experiments were performed at 32 °C in a standard or perforated patch whole cell configuration. Recordings were made using an EPC-10 amplifier and Pulse software. The action potential-like voltage-clamp commands were constructed by averaging 12 and 15 action pontential in EndoC-βH1 and EndoC-βH2, respectively and used to elicit currents. Action potentials were also recorded from human primary beta cells to allow comparison with action potential shape in the two cell lines.

For the perforated patch recordings and measurements of K^+^ current, the extracellular medium was composed of (mmol/L) 138 NaCl, 3.6 KCl, 0.5 MgSO_4_, 0.5 NaH_2_PO_4_, 5 NaHC0_3_, 1.5 CaCl_2_ and 5 HEPES (pH 7.4 with NaOH). The extracellular medium was supplemented with 1, 6 or 20 mmol/L glucose and tolbutamide (0.2 mmol/L) and diazoxide (0.5 mmol/L) as indicated. For the measurements of Na^+^ and Ca^2+^ currents and capacitance measurement of exocytosis, outward K^+^ currents were inhibited by inclusion of 20 mmol/L tetraethylammonium (TEA) in the extracellular medium (NaCl correspondingly reduced to maintain iso-osmolarity). Na^+^ currents were recorded after substitution of CaCl_2_ for CoCl_2_ (to block the voltage-gated Ca^2+^ currents).

Various pipette-filling media were used. For the perforated patch measurements, the pipette-filling medium contained (mmol/L) 128 K-gluconate, 10 KCl, 10 NaCl, 1 MgCl_2_ and 10 HEPES (pH 7.35 adjusted with KOH). Perforation of the membrane was achieved using amphotericin B (0.24 mg/ml)^24^. For the capacitance measurements of exocytosis, the intracellular medium contained (mmol/L) 129 CsOH, 125 Glutamic acid, 20 CsCl, 15 NaCl, 1 MgCl_2_, 0.05 EGTA, 3 ATP, 0.1 cAMP, 5 HEPES (pH7.2 with CsOH). For recordings of the voltage-gated K^+^ currents, the pipette was filled with (mmol/L) 120 KCl, 1 MgCl_2_, 1 CaCl_2_, 3 MgATP, 10 EGTA, 10 HEPES (pH 7.15 with KOH). A similar medium was used for the Na^+^ and Ca^2+^ currents measurements except that KCl was equimolarly replaced by CsCl and pH adjusted with CsOH.

#### Current analysis

The net contribution of a current component to the total current was estimated by subtracting the current responses after addition of specific blockers from those observed prior to the addition. In experiments involving multiple blockers, these were applied sequentially in the continued presence of the other inhibitor(s). To compensate for variations in cell size, responses have been normalised to cell capacitance, which is proportional to cell surface area. Compared to primary human beta-cells, both cell lines appear more fragile and it was difficult to maintain the recordings long enough to allow sequential application of multiple channel blockers.

#### Sodium channels activation/inactivation

The voltage dependence of activation by fitting to the Boltzmann equation (Eq. 1) the increased conductance normalised to its maximum evoked from a voltage of −70 to 20 mV:

1 $$y=1-\frac{1}{1+\exp (\frac{x-V0.5}{dx})}$$

where *V*_0.5_ is the membrane potential at which activation is half-maximal and *dx* is the slope.

Monophasic inactivation of the Na^+^ current was fitted to a single Boltzmann equation (Eq. 2):

2 $$y=1-\frac{1}{1+\exp (\frac{x-V{\rm{h}}}{dx})}$$

where *V*_h_ is the voltage at which current inactivation is half-maximal, and x is the slope factor.

### [Ca^2+^] imaging

The cells were plated at the same density as that in the culture flask but in a 60 μl strip on the coverslip. EndoC-βH1 and –βH2 cells were transfected with GCaMP5G, expressed from pCMV-GCaMP5G (Addgene plasmid # 31788) using lipofectamine (ThermoFischer Scientific, Loughborough) as detailed in the Supplementary material. Prior to experiments, the coverslip was transferred to an in-house recording chamber, superfused at a rate of 60 μl/min with KRB solution at 34 °C and imaged using a 10–14x magnification on a Zeiss AxioZoom.V16 microscope (Zeiss, Germany). Synchronisation of the recording with the perfusion is achieved by addition of food colorant to the superfusion medium at the end of the experiment. Image sequences were analysed as detailed in the Supplementary material.

### Immunocytochemistry

EndoC-βH1 and -βH2 were fixed in 4% paraformaldehyde, permeabilised in 0.1% (v/v) Triton X-100, and blocked in 5% (w/v) goat serum. Immunofluorescence was performed using a guineapig anti-insulin antibody directed against human B chain (in-house, 1:1000) and anti-guineapig Texas red secondary antibody. Glucagon and somatostatin were labeled using anti-mouse glucagon antibody (Sigma, 1:1000) and anti-mouse somatostatin antibody (Santa Cruz Biotechnology, Germany, 1:200). Both were detected using an anti-imouse FITC secondary antibody and mounted in Vectashield Mounting Medium with DAPI (Vector Laboratories). Cells were imaged using an LSM 510 META confocal laser scanning module arranged on an Axiovert 200 microscope and a Plan-Apochromat 63x/1.4 oil immersion objective (Carl Zeiss). An argon laser was used to excite at λ = 488 nm and a HeNe laser was used to excite Texas red at λ = 543 nm. DAPI was excited in two-photon mode using the 740 nm line of an infrared light Chameleon laser. Proportion of SST/GLC positive cells were determined using FIJI software.

### Electron microscopy

EndoC-βH1 and -βH2 cells cultured either in flasks or on a 0.4 μm polycarbonate membrane (Nunc, #137052) were fixed in 2.5% glutaraldehyde, post-fixed in 2% uranyl acetate, dehydrated in graded methanol, and embedded in London Resin Gold (Agar Scientific, Stansted, UK). Ultrathin sections (70 nm) cut onto nickel grids were immunolabelled with anti-glucagon (Sigma, 1:100) or anti-somatostatin (Santa Cruz Biotechnology, #25262, 1:10) followed by anti-rabbit biotin (Vector Laboratories, Peterborough) and streptavidin gold 15 nm (British Biocell International, Cardiff, UK). Insulin was immunolabelled (DAKO, Ely, UK, 1:500) followed by anti-guinea pig gold 10 nm (British Biocell International). Sections were viewed on a Joel 1010 microscope (accelerating voltage 80 kV) with a digital camera (Gatan, Abingdon). The analysis is detailed in the Supllementary material.

### Morphology

The granule density (*N*_A_) can be converted to the volume density (*N*_*V*_) using the equation (Eq. 3):

3 $${N}_{V}=\frac{{N}_{A}}{(T+D-2h)}$$

where *D* is the measured averaged granule diameter, *h* is the smallest granule diameter and *T* the section thickness. From the average vesicle area, the average diameter (*D*) was estimated as 0.2 μm. The smallest vesicle diameter detected was 0.025 µm (h) which corresponds to Nv values of ~13 and 20 vesicles per μm^3^ in EndoC-βH1 and βH2, respectively.

### RNA sequencing

For both cell lines, RNA was extracted using TRIzol and sequenced at the Oxford Genomics Centre (Wellcome Centre for Human Genetics, University of Oxford)^25,26^. These data are deposited at the European Nucleotide Archive (https://www.ebi.ac.uk/ena) under the accession number PRJEB23293 (a summary of the transcriptome analysis can also be found in Supplementary dataset). Details are found in the Supplementary material. Gene counts were quantified using featureCounts v1.5.0-p2)^26^, and converted into TPM (Transcripts Per kilobase Million).

### Data analysis

Data are presented as mean ± SEM. The number of experiments (n) and details of the statistical analysis are in the figure legends. P values are in the corresponding section in the results. For electrophysiology, calcium imaging, microscopy, n are representative of the number of cells from several passages of the same batch. For insulin secretion assay, n are representative of independent experiments performed at different passages from at least two different batches of cells. For each hormone secretion experiment, stimulations were performed in technical triplicates.

## Results

### Glucose-induced insulin release in EndoC-βH1 and -βH2 cells

**Figure 1:**
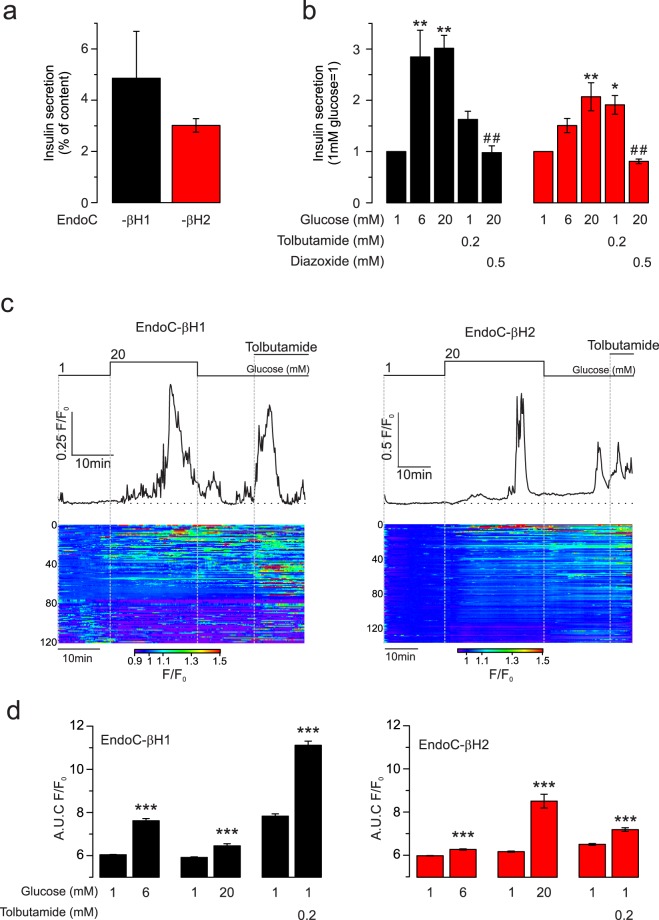
Glucose-induced insulin secretion and [Ca^2+^]_i_ in EndoC-βH1 and -βH2 cells. (**a**) Basal insulin secretion elicited by 1 mmol/L glucose for 1 hour normalised to insulin content in EndoC-βH1 (black) and -βH2 (red). n = 3 independent experiments. (**b**) Insulin secretion normalised to basal stimulated during 40 min at 1, 6 or 20 mmol/L glucose and tolbutamide or diazoxide as indicated (n = 3). * and ^#^p < 0.05, comparison to 1 mmol/L and 20 mmol/L glucose respectively, ** and ^##^p < 0.01 (ANOVA and Tukey). (**c**) Representative [Ca^2+^]_i_ responses (normalised to initial fluorescence; F/F_0_) to 20 mmol/L glucose and 0.2 mmol/L tolbutamide, EndoC-βH1 (left) and -βH2 (right). Shown below are heatmaps from ≥120 cells from the same session (synchronized to representative traces) and displayed as F/F_0_ (colour scale at the bottom). (**d**) Quantification of the area under the curve (AUC) per minute for each condition over 4 and 3 sessions for EndoC-βH1 and -βH2 respectively; ***p < 0.001, paired t-test.

Insulin secretion at 1 mmol/L glucose (normalised to insulin content) was ~4.5% per hour (h) and ~3%/h in EndoC-βH1 and -βH2 cells (n = 3 independent experiments), respectively (Fig. 1a). Increasing glucose to 6 mmol/L, stimulated insulin secretion by 3-fold in EndoC-βH1 (p = 0.006) and by 1.5-fold in the EndoC-βH2 cells (p = 0.242) (Fig. 1b). Whereas elevating glucose to 20 mmol/L had no additive effect in EndoC-βH1 (p = 0.003), it resulted in a further 50% stimulation in EndoC-βH2 cells (p = 0.006). In both cell types, the stimulatory effect of glucose was prevented by the K_ATP_ channel activator diazoxide (0.5 mmol/L) (EndoC-βH1 p = 0.003, EndoC-βH2 p = 0.002) and mimicked (in part) by the K_ATP_ channel blocker tolbutamide (0.2 mmol/L) (EndoC-βH1 p = 0.52, EndoC-βH2 p = 0.016) (Fig. 1b).

Transcriptomic analysis of these cell lines obtained by RNA-sequencing revealed that key genes involved in the beta-cell glucose sensing are expressed in these cell lines. The K_ATP_ channels subunits *KCNJ11* (Kir6.2) and *ABCC8* (SUR1) as well as *SLC2A1* (encoding for the glucose transporter GLUT1) and *GCK* (glucokinase) are expressed at comparable levels in EndoC-βH1 and -βH2 (Supplementary Fig. 1 and Supplementary dataset). Whilst *SLC2A2* (GLUT2) is expressed at levels approaching *SLC2A1* in EndoC-βhH1 cells, its expression is very low in EndoC-βH2 cells (Supplementary Fig. 1 and Supplementary dataset).

### Glucose triggers increases in [Ca^2+^]_i_ in EndoC-βH1 and -βH2 cells

Insulin is secreted in response to an elevation in cytoplasmic Ca^2+^ ([Ca^2+^]_i_). We correlated the insulin secretion data to changes in [Ca^2+^]_i_ in cells transfected with the genetically encoded Ca^2+^ indicator GCaMP5 (Fig. 1c). The heatmaps below the traces (GCamp5 fluorescence normalised to basal fluorescence; F/F_0_) illustrate the substantial heterogeneity between cells. Basal activity was higher in EndoC-βH1 than in EndoC-βH2 cells. Spontaneously active cells responded poorly to both high glucose and tolbutamide (bottom part of the heatmap). However, despite the variability, significant [Ca^2+^]_i_ increases were evoked by both 6 mmol/L (n = 523 cells p = 7.6 × 10^−47^ and n = 225 cells p = 9.2 × 10^−22^) and 20 mmol/L glucose (n = 442 cells p = 1.7 × 10^−9^ and n = 236 cells p = 3.1 × 10^−13^) or 200 µmol/L tolbutamide (n = 965 cells p = 6.5 × 10^−88^ and n = 461 cells p = 3.0 × 10^−19^) in each cell line (for EndoC-βH1 and -βH2 respectively) (Fig. 1d).

### EndoC-βH1 and -βH2 cells are electrically excitable

**Figure 2:**
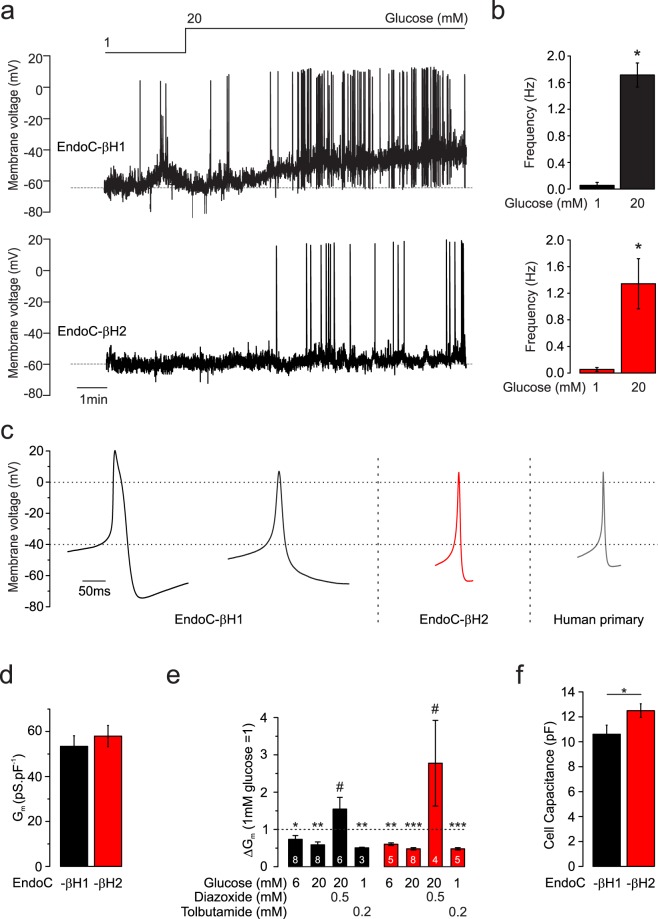
K_ATP_ channel activity and action potential firing. (**a**) Representative membrane potential recording made in EndoC-βH1 (top) and EndoC-βH2 (lower) at 1 and 20 mmol/L glucose (as indicated). (**b**) Action potential frequency in EndoC-βH1 and -βH2 cells at 1 and 20 mmol/L glucose. *p < 0.05 (1 mmol/L glucose n = 5 cells each, 20 mmol/L glucose n = 3 and 4 cells for EndoC-βH1 and -βH2, respectively). Student’s t-test. (**c**) Examples of average action potentials (AP) recorded in two EndoC-βH1 cells, and EndoC-βH2 cell and a primary human beta-cell as indicated (average of at least 12 and 15 APs from the same recording). (**d**) Resting K_ATP_ channel activity in EndoC-βH1 and -βH2 cells measured at 1 mmol/L glucose (n = 22 and 21 cells). (**e**) Effects of glucose, tolbutamide and diazoxide (added at the indicated concentrations) on whole-cell K_ATP_ channel activity. K_ATP_ channel activity has been normalised to that at 1 mmol/L glucose. *vs. 1 mmol/L and ^#^vs. 20 mmol/L glucose. * and ^#^p < 0.05, **p < 0.01, and ***p < 0.001, paired t-test. Number of cells (n) is inserted in the corresponding columns. (**f**) Cell capacitance in EndoC-βH1 and -βH2 cells *p < 0.05 (n = 30 and 32 cells respectively). Student’s t-test.

We correlated insulin secretion and changes in [Ca^2+^]_i_ with electrical activity. At 1 mmol/L glucose, the membrane potential was −70 to −60 mV and many EndoC-βH1 and -βH2 cells exhibited spontaneous action potential firing. Increasing glucose concentrations to 20 mmol/L resulted in membrane depolarisation to −55 mV and stimulation of electrical activity (Fig. 2a). On average, glucose (20 mmol/L) increased action potential frequency by 30- (n = 3 cells, p = 0.03) and 25-fold (n = 4 cells, p = 0.01) in EndoC-βH1 and -βH2 cells, respectively (Fig. 2b). The characteristics of glucose-induced action potentials in EndoC-βH2 were largely similar to those in primary human beta-cells potential and peaked at 5 mV (Fig. 2c). However, in EndoC-βH1 cells, the action potentials were generally broader and in 90% of the measured cells, they peaked at voltages above +5 mV.

The resting membrane conductance (G_m_) averaged 50–60 pS/pF (EndoC-βH1: n = 21 cells; EndoC-βH2: n = 22 cells) in both cell types (Fig. 2d). These values are in good agreement with membrane conductance measured in primary human beta-cells^1^. High glucose and tolbutamide reduced G_m_ by ≈50% (EndoC-βH1: n = 8 cells, p = 0.003 and n = 3 cells p = 0.001; EndoC-βH2: n = 8 cells, p = 6.4 × 10^−7^ and n = 5, p = 8.63 × 10^−5^, respectively) (Fig. 2e). Conversely, addition of the K_ATP_ channel activator diazoxide (0.5 mmol/L) in the presence of 20 mmol/L glucose increased G_m_ by 200–400% (EndoC-βH1: n = 6 cells, p = 0.01; EndoC-βH2: n = 4 cells, p = 0.01). The average cell capacitance (proportional to the cell size) is of 10.6 pF and 12.5 pF for EndoC-βH1 and -βH2 (n = 30 and 32 cells, p = 0.04), respectively (Fig. 2f).

### Voltage-gated channels

We next characterised the three voltage-gated membrane currents (Na^+^, Ca^2+^ and K^+^) that underlie action potentials.

#### Na^+^ channels

**Figure 3:**
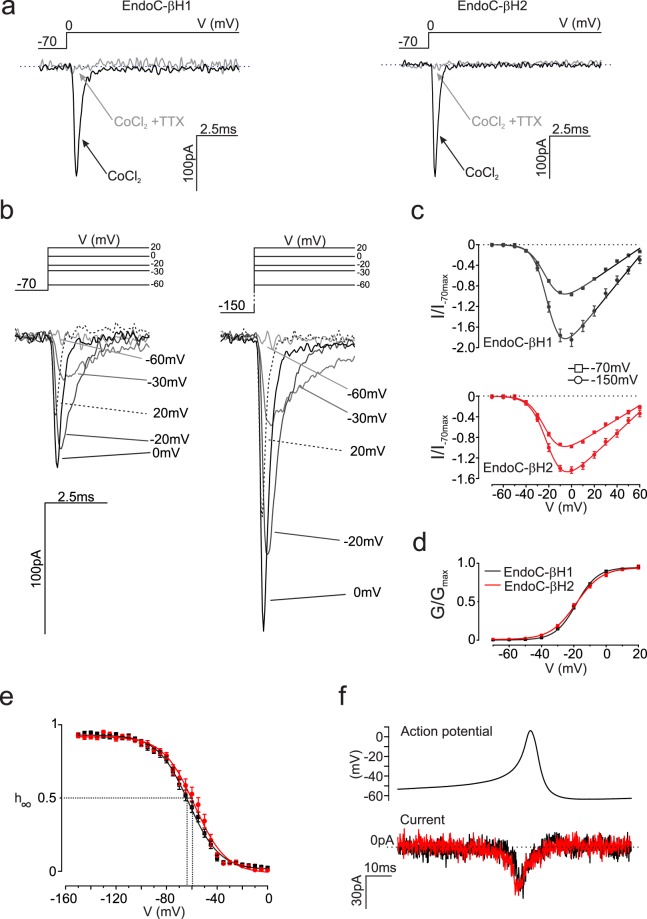
Voltage-gated Na^+^ currents. (**a**) Voltage-gated Na^+^ currents (I_Na_) in EndoC-βH1 (left) and -βH2 cells (right) in the presence of Co^2+^ (1 mmol/L) with or without TTX (0.1 μg/ml; as indicated) during depolarisations from −70 to 0 mV (representative of 24 and 11 cells for EndoC-βH1 and -βH2 respectively). (**b**) Families of I_Na_ recorded during depolarisations from −70 (left) and −150 mV (right) during depolarisations to indicated membrane potentials. (**c**) I_Na_ current-voltage relationships recorded from a holding potential of −70 or −150 mV in EndoC-βH1 (upper panel; n = 24 cells) and -βH2 cells (lower panel; n = 11 cells). Current responses normalised to the peak current elicited by a depolarisation from −70 mV to 0 mV. (**d**) I_Na_ activation when cells were held at −70 mV in EndoC-βH1 and -βH2 cells. Curves were derived by fitting Eq. 1. to current responses of individual experiments (n = 26 − 23 cells). (**e**) Steady-state voltage-dependent inactivation of I_Na_ in EndoC-βH1 (red) and -βH2 cells (black) estimated from a two-pulse protocol: a standard test pulse to 0 mV was preceded by 50 ms conditioning pulse to membrane potentials between −150 and 0 mV (n = 26 and 32 cells). Data have been normalised to the maximum current during the test pulse (h_∞_ = I/I_max_). Responses have been fitted to Eq. 2. (**f**) I_Na_ evoked by action potential-like voltage-clamp commands in EndoC-βH1 (black; n = 26 cells) and -βH2 cells (red; n = 32 cells).

In both cell lines, voltage-gated Na^+^ currents (I_Na_) elicited by membrane depolarisations from −70 to 0 mV were insensitive to the Ca^2+^ channel blocker Co^2+^ but highly blocked by tetrodotoxin (TTX, a blocker of most voltage-gated sodium channels) (Fig. 3a). Figure 3b shows families of Na^+^ currents during depolarisations between −60 and +20 mV evoked from a holding potential of −150 mV or −70 mV. Both activation and inactivation became more rapid with stronger depolarisations. At 0 mV, activation and inactivation were complete within <1 ms and 5 ms, respectively. Figure 3c shows the current (I)-voltage (V) relationships recorded when the cells were held at either −70 mV or −150 mV. In both cell types, I_Na_ became detectable during depolarisations to −40 mV, was maximal at 0 mV and decreased at more positive voltages with an extrapolated reversal potential at +70 mV. The maximum current elicited from −70 mV (−9.5 ± 1 pA/pF, n = 24 EndoC-βH1 cells and −19 ± 7 pA/pF, n = 11 EndoC-βH2 cells) increased by 90% and 60% respectively when the cells were held at −150 mV (Fig. 3c). This suggests that the Na^+^ channels in EndoC-βH1 and –βH2 cells undergo partial voltage-dependent steady-state inactivation at unphysiologically negative membrane potentials, in agreement with what is observed in other insulin-secreting cells^27^.

We described the voltage dependence of activation from −70 mV by fitting the I–V relationship to Eq. 1. Activation of I_Na_ was half-maximal (V_0.5_) at −18 ± 0.3 mV (n = 26) and −19 ± 0.4 mV (n = 23) in EndoC-βH1 and EndoC-βH2 cells, respectively (Fig. 3d).

Voltage-gated Na^+^ channels characteristically undergo voltage-dependent inactivation. We characterised this by a two-pulse protocol (a standard test pulse was preceded by a conditioning prepulse). In EndoC-βH1 and -βH2 cells 58% and 36% of the measured I_Na_ exhibited monophasic inactivation; half-maximal inactivation (*V*_h_; see Eq. 2 in Supplementary material) was at −63 ± 2 mV (n = 15) and −59 ± 2 mV (n = 12) in EndoC-βH1 and -βH2 cells, respectively (Fig. 3e). The remaining cells showed biphasic voltage dependence of inactivation and in addition to the main component contained a current component that accounted for 30–40% of the total current that inactivated with *V*_h_-values of −94 ± 4 mV (n = 11) to −91 ± 3 mV (n = 21) (not shown). The latter component likely accounts for the increase in I_Na_ amplitude when the cells were held at −150 mV rather than −70 mV, but its functional significance remains obscure, as it will be completely inactivated at physiological membrane potentials. Overall, the I_Na_ activation and inactivation properties in EndoC-βH1 and -βH2 cells are similar to those observed in primary human beta-cells^23,28^.

Figure 3c shows I_Na_ during an action potential-like voltage-clamp stimulation (based on the action potentials recorded in EndoC-βH2 cells). Peak I_Na_ elicited by this stimulation paradigm were small: 3.1 ± 0.3 pA.pF^−1^ and 3.9 ± 1 pA.pF^−1^ in EndoC-βH1 and -βH2 cells, respectively (Fig. 3f).

At the mRNA level and compared to the gene set of Na^+^ channels transcripts EndoC-βH1 and -βH2 cells express particularly high levels of *SNC8A* (Na_v_1.6) and *SNC9A* (Na_v_1.7). *SCN3A* (Nav1.3) is also highly expressed in EndoC-βH2 cells. Furthermore, *SCN*5*A* and *SCN7A* are expressed at relatively high levels but these encode TTX-resistant channels. Given that I_Na_ in both cell types is TTX-sensitive, it appears that these transcripts do not give rise to functional channels. Of the β-subunits, only *SCN3B* is expressed at significant levels (Supplementary Fig. 2, and Supplementary dataset).

#### Ca^2+^ channels

**Figure 4:**
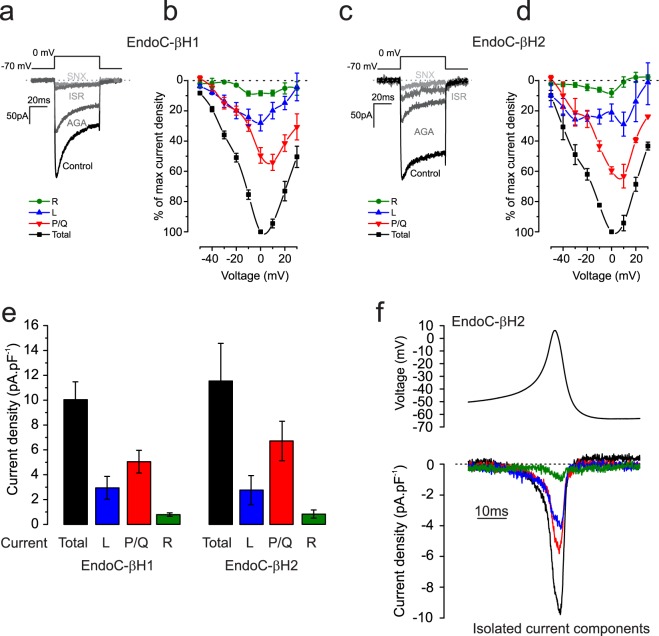
Voltage-gated Ca^2+^ currents. (**a**,**c**) Voltage-gated Ca^2+^ currents (I_Ca_) evoked by membrane depolarisations between −70 and 0 mV under control conditions and following sequential addition of ω-agatoxin (200 nmol/L), isradipine (10 μmol/L) and SNX482 (100 nmol/L) in EndoC-βH1 (**a**) and EndoC-βH2 (C). (**b,d**) Current voltage relationships normalised to maximum peak total current in each cell for the total I_Ca_ and the isolated P/Q (ω-agatoxin-sensitive), L- (isradipine-sensitive) and R-type (SNX482-sensitive) components in EndoC-βH1 (**b**) and EndoC-βH2 (**d**). (**e**,**f**) Current density (peak amplitude normalised to cell capacitance) histograms showing the maximum total as well as pharmacologically isolated R-, L- and P/Q-type Ca^2+^ current components in EndoC-βH1 (**e**) and EndoC-βH2 (**f**). (**g**) I_Ca_ evoked in EndoC-βH2 cells using a voltage-clamp command based on action potentials recorded in these cells. The traces shown correspond to the total I_Ca_ as well as the isolated P/Q-, L- and R-type components. Data are based on measurements in 5 and 3 cells for EndoC-βH1 and -βH2 respectively.

Voltage-gated Ca^2+^ currents (I_Ca_) were recorded in the presence of TTX. The relative contribution of P/Q-, L- and R-type Ca^2+^ channels was estimated by sequential addition of ω-agatoxin, isradipine and SNX482 in presence of TTX to inhibit I_Na+_(Fig. 4a,c). Figure 4b,d compares the voltage dependence of the total I_Ca_ and the different (pharmacologically isolated) I_Ca_ components (EndoC-βH1, n = 5; EndoC-βH2, n = 3 cells). In both cell lines, there was a ‘shoulder’ at voltages between −50 and −20 mV. In both cell types, P/Q-type Ca^2+^ channels accounted for ~60% of the total current, whereas L- and R-type Ca^2+^ channels contributed 20–30% and 5–10%, respectively (Fig. 4b,d). Similar to previous measurements on primary human beta-cells^23^, the total I_Ca_ density was ~10 pA/pF in both cell types (Fig. 4e). Figure 4g compares the activation of the total I_Ca_ and the respective components during an action potential in EndoC-βH2 cells. It is clear that both the L- and P/Q-type Ca^2+^ channels activate during the brief action potential.

Consistent with electrophysiology, transcriptomic profiling revealed high expression of *CACNA1A* (P/Q-type) and *CACNA1C*/*D* (L-type) Ca^2+^ channels and low levels of *CACNA1E* (R-type) expression. Moreover, *CACNA1H* (T-type) Ca^2+^ channels are expressed at high levels and are likely to account for the shoulder between −50 and −20 mV in the current-voltage relationships (Fig. 4a,c). Of the auxiliary subunits, high expression of *CACNG4* (γ_4_), *CACNA2D* (α_2_δ_2_) and *CACNB1-3* (β_1_-β_3_) was observed (Supplementary Fig. 3; and Supplementary dataset).

#### K^+^ channels

**Figure 5:**
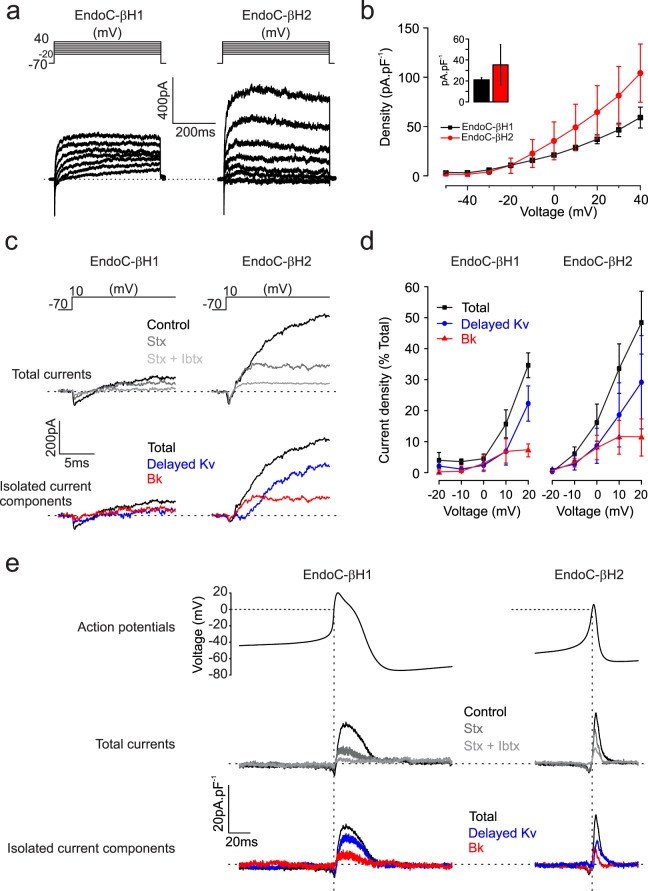
Voltage-gated K^+^ currents. (**a**) Families of voltage-gated K^+^ currents (I_K_) in EndoC-βH1 (left) and EndoC-βH2 (right) during voltage steps from −70 mV to membrane potentials between −20 and +40 mV. (**b**) Current density-voltage relationships in EndoC-βH1 (black) and EndoC-βh2 (red). Current density recorded in both cell lines at 0 mV, inset. (**c**) I_K_ recorded during membrane depolarisations to 10 mV under control conditions, after addition of stromatoxin (Stx, 100 nmol/L) and iberiotoxin (Ibtx, 100 nmol/L) as indicated. Bottom panels show pharmacologically isolated K_V_2 (Stx-sensitive) and BK Ibtx-sensitive) current components. (**d**) Voltage dependence of K_V_2 and BK currents. Responses have been normalised to peak total I_K_ at +50 mV. (**e**) I_K_ evoked in EndoC-βH1 and -βH2 cells using voltage-clamp commands based on the action potentials recorded in the respective cells under control conditions and the isolated Stx- and Ibtx-sensitive components. Data are based on measurements in n = 4 EndoC-βH1 cells and n = 3 EndoC-βH2 cells.

Voltage-activated K^+^-currents (I_K_) elicited during membrane depolarisations from −70 mV to membrane potentials between −20 and +70 mV in EndoC-βH1 and -βH2 cells are shown in Fig. 5a. The amplitude of I_K_ increased linearly with the applied voltage and the density was ~50% larger in EndoC-βH2 (n = 3) than in -βH1 (n = 4 cells) (Fig. 5b). We estimated the contribution of voltage- (K_v_2) and Ca^2+^-activated (BK) channels to I_K_ by sequential addition of their cognate blockers stromatoxin and iberiotoxin respectively (Fig. 5c). Notably, BK channels played a more prominent role in EndoC-βH2 than in EndoC-βH1 during depolarisations to −10 and 0 mV (Fig. 5d). Figure 5e shows the contribution of K_V_2 and BK channels to the outward current during voltage-clamp depolarisations based on the action potentials in EndoC-βH1 and -βH2 cells. This analysis reveals that BK current component activated rapidly during the action potential and at a time when no outward current was seen in the EndoC-βH1 cells (Fig. 5e, vertical dashed line).

The transcript profile of the K channels expressed in EndoC-βH1 and -βH2 cells reveals high levels of the K_V_2 channel genes *KCNH6*, *H2*, *KCNB2, KCNQ2* and the BK channel genes *KCNMA1* and *KCNMB3* (Supplementary Fig. 4 and Supplementary dataset).

### Exocytosis

**Figure 6:**
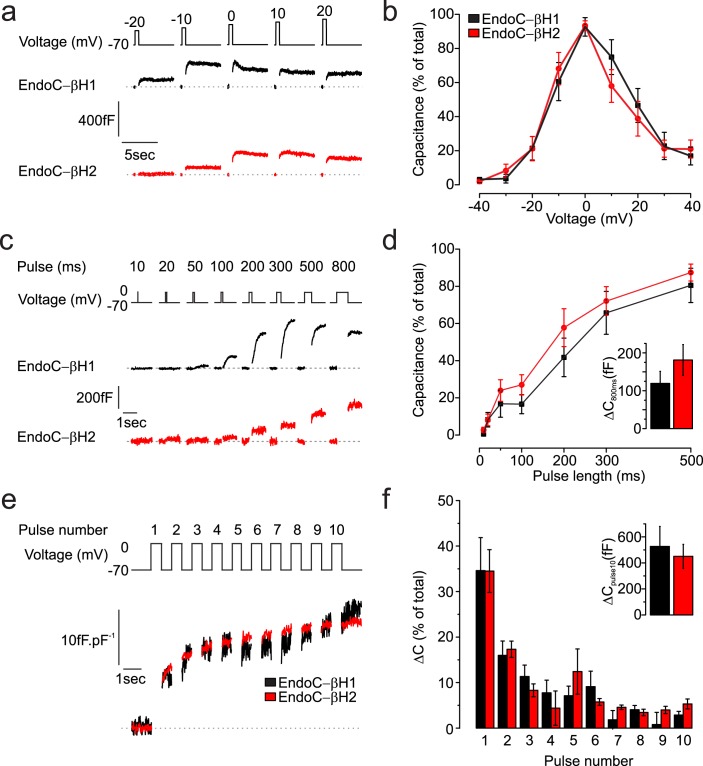
Voltage dependence and kinetics of exocytosis. (**a**,**b**) Exocytosis evoked by 500 ms depolarisations from −70 mV to indicated membrane potentials (top) in EndoC-βH1 (black) and EndoC-βH2 cells (red). (**b**) Voltage dependence of exocytosis in EndoC-βH1 (black; n = 6 cells) and EndoC-βH2 cells (red; n = 10 cells). Responses are normalised to maximum response. (**c**,**d**) Exocytosis evoked by progressively longer (10–800 ms) depolarisations from −70 to 0 mV. (**d**) Relationship between pulse duration and exocytotic response in EndoC-βH1 (black; n = 12 cells) and EndoC-βH2 cells (red; n = 14 cells). Exocytosis is normalised to the response evoked by the 800 ms depolarisation (inset). (**e**,**f**) Representative trace of exocytosis evoked by trains of ten 500 ms depolarisations from −70 to 0 mV in EndoC-βH1 (black) and EndoC-βH2 cells (red). (**f**) Exocytotic response during the individual depolarisations in EndoC-βH1 (black, n = 12 cells) and EndoC-βH2 cells (red, n = 14 cells). Responses are normalised to the total increases in cell capacitance evoked by the trains in individual cells (inset).

Depolarisation-evoked exocytosis was monitored as increases in cell capacitance. Figure 6a,b compares the voltage dependence of exocytosis. Exocytosis was trigged by 500 ms depolarisations to voltages between −40 and +40 mV. In both cell types, exocytosis was elicited at voltages above −30 mV, was maximal at ~0 mV and declined at more depolarised voltages (EndoC-βH1, n = 6; EndoC-βH2, n = 10 cells). This voltage dependence echoes that of I_Ca_. The kinetics of exocytosis during depolarisation to 0 mV (i.e. close to the peak of the action potential) was determined by application of progressively longer depolarisations (Fig. 6c,d). In both EndoC-βH1 and -βH2 cells, exocytosis was elicited by depolarisations as short as 20 ms, showed an initial plateau between 50 and 100 ms and a secondary acceleration during longer depolarisations (EndoC-βH1 n = 12; EndoC-βH2 n = 14 cells). The exocytotic response to a 800 ms depolarisation once the capacitance had stabilized was 119 ± 31 fF and 181 ± 40 fF in EndoC-βH1 and -βH2 respectively (Fig. 6d, insert).

We also monitored exocytosis during repetitive stimulation consisting of ten 500 ms depolarisations to 0 mV (Fig. 6e). Figure 6f compares the responses to the individual depolarisations in EndoC-βH1 (n = 12 cells) and -βH2 (n = 14 cells). The kinetics of exocytosis was biphasic, echoing the kinetics observed in primary human beta-cells^29^. The largest response was elicited by the initial depolarisations with the first two pulses accounting for ≥50% of the total increase in capacitance and the responses during the last four pulses were small. The total increase in cell capacitance evoked by the train averaged 450 fF in both cell types (Fig. 6f, inset).

Transcriptomic analysis of genes encoding proteins involved in exocytosis revealed higher expression of *SNAP25* in EndoC-βH1, than in EndoC-βH2 cells. The other SNAREs, *VAMP2* and Syntaxin1A (*STX1A*) were expressed to the same extent (Supplementary Fig. 5 and Supplementary dataset). Other components of the exocytotic machinery, including munc18a-c (*STXBP1-3*), munc13b (*UNC13B*) and the complexins (*CPLX1-2*) were also highly expressed. Of the Ca^2+^ sensors of exocytosis, the high-affinity Ca^2+^ sensor *SYT7* was the predominant synaptotagmin in both cell types but relatively high levels of *SYT1*, *5* and 9 were also detected in EndoC-βH1 cells.

### Subset of EndoC-βH1 and -βH2 cells are polyhormonal cells

**Figure 7:**
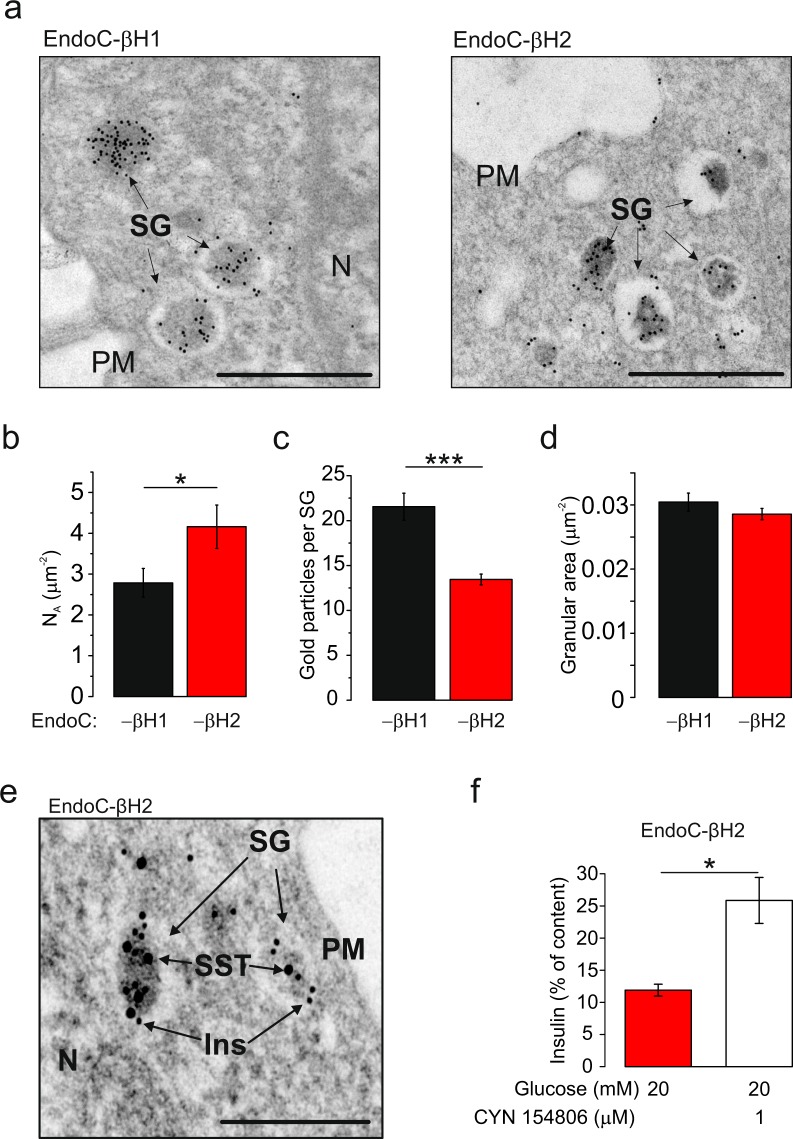
Ultrastructural analysis. (**a**) Electron micrograph of immunogold- labeled insulin (15 nm gold particles) in EndoC-βH1 (left) and EndoC-βH2 cells (right). Abbreviations: SG, Secretory Granule; PM, Plasma Membrane; N, Nucleus. Scale bar: 1 µm. (**b**–**d**) Quantification of the granule density (N_A_, number of granule per cytoplasmic area; (**b**), number of immunogold particles per SG (**c**) and granule cross-sectional area (**d**). Data in (**a**–**d**) representative for 10 cells of each type.*p < 0.05 and ***p < 0.001 (Student’s t-test) (**e**) Some EndoC-βH2 cells have SG containing both insulin (10 nm gold particles) and somatostatin (15 nm gold particles). See also Supplementary Figure S6. Abbreviations: Ins, insulin; SST, somatostatin. Scale bar: 0.25 µm. (**f**) Potentiation of insulin secretion at 20 mmol/L glucose by the SSTR antagonist CYN154806 (1 µM). *P < 0.05, Student’s t-test (n = 3 independent experiments).

Ultrastructural analysis revealed that the secretory granules in EndoC-βH1 and -βH2 were of variable size and that most granules exhibited a clear thin halo and a central insulin-containing core (Fig. 7a). The granule density (granules per cytoplasm area; N_A_) was slightly higher in EndoC-βH2 cells than in the -βH1 cells (Fig. 7b, n = 10 cells in both cell types, p = 0.045) but the intragranular insulin density (estimated from immunogold labelling) was correspondingly reduced (p = 9.1 × 10^−8^) (Fig. 7c). On average, 4.5 ± 1.7% (EndoC-βH1) and 7.6 ± 2.4% (EndoC-βH2) of the vesicles per EM section were directly docked to the plasma membrane. In both cell types, the cross-sectional granule area was 0.03 µm2 (Fig. 7d; n = 265 and 337 vesicles in EndoC-βH1 and -βH2 respectively), corresponding to a granule diameter of 200 nm from which we estimate a granule surface area of 0.12 µm^2^ (assuming spherical geometry).

Immunocytochemistry suggested that most EndoC-βH1 and -βH2 cells contained only insulin-labelled granules but that a small subset of cells also contained somatostatin- and/or glucagon-positive granules (Supplementary Fig. 6). Whereas insulin- and glucagon-positive cells were found only in EndoC-βH2, somatostatin-positive cells were found in both cell lines. Electron microscopy in conjunction with immunogold labelling indicated that somatostatin and insulin could be contained in the same granules (Fig. 7e). Transcript analysis confirmed high expression of insulin (*INS*) in both EndoC-βH1 and -βH2 cells and that glucagon (*GCG*) and somatostatin (*SST*) were expressed at particularly high levels in EndoC-βH2 cells. There was also high expression of the somatostatin receptors 1, 2 and 3 (*SSTR1-3*) (Supplementary Fig. 7 and Supplementary dataset). Notably, addition of the SSTR2 antagonist CYN154806 doubled glucose-induced insulin secretion in EndoC-βH2 cells (p = 0.035) (Fig. 7f).

### Transcriptome and foetal origin

The relative low stimulation of insulin secretion and the polyhormonality observed in EndoC-βH2 cells may be remaining features from their foetal origin^30,31^. To address this question, we compared the publically available transcriptomic data from foetal and adult human pancreatic beta cells with the RNA sequencing datasets we generated from EndoC-βH1 and -βH2 cell lines. We analysed a subset of genes that were consistently lowly or not expressed in human adult beta cells but highly expressed in foetal beta cells^32,33^. Such genes encoded for either components of the early development (*NGN3* (Neurogenin 3)) or the inflammatory response (*PTGS2* (Prostaglandin-Endoperoxide Synthase 2), *S100A9* (S100 Calcium Binding Protein), and *DEFA4* (Defensin Alpha 4))^32^. Comparison of a subset of genes known to be mainly expressed in human foetal beta cells revealed that the human beta cell lines’ transcriptomes are closer to the one of an adult primary beta cell (Supplementary Fig. 9).

## Discussion

We have characterised the electrophysiological properties of two human beta-cell lines: EndoC-βH1 and -βH2. We confirmed that both cell lines are glucose-responsive with the stimulatory effect on insulin secretion limited to a 2- to 3-fold enhancement^8–10^. At the transcriptional level, these cell lines exhibit a greater similarity to adult than to foetal human beta cells. We acknowledge that these human beta-cell lines exhibit some variability between different batches in terms of glucose responsiveness of insulin secretion and even presence of ion channels. However, this is not unique to these cell lines and, provided appropriate controls are performed in parallel, this variability should be possible to manage. In this context it is worth remembering that also primary human islets show significant inter-preparation variability. Here we discuss a few aspects of this work that we find particularly interesting and important. We correlate our findings in the EndoC-βH1 and EndoC-βH2 cell lines to what has previously been found for primary human beta-cells.

### K_ATP_ channel activity and action potential firing

The action potentials induced by glucose originated from a membrane potential of ~−50 mV and usually peaked at +5 mV (or above). Both EndoC-βH1 and -βH2 are equipped with K_ATP_ channels. The measured K_ATP_ channel density at 1 mmol/L glucose in metabolically intact beta-cells is 0.05 nS/pF. As the cells had cell capacitances of 10–12 pF, these values correspond to a resting conductance of ~0.5 nS and an input resistance (the reciprocal of the resting conductance) as high as 2 GΩ. The former value is only ~10% of the corresponding value in mouse beta-cells but comparable to that observed in primary human beta-cells^1^. The high input resistance suggests that small currents (such as those resulting from the opening of individual ion channels) may produce a membrane depolarisation large enough to trigger an action potential. This probably explains why EndoC-βH1 and -βH2 cells generate spontaneous action potentials and exhibit [Ca^2+^]_i_ transients at 1 mmol/L glucose.

Both increasing glucose (to 6 and 20 mmol/L) and application of the K_ATP_ channel inhibitor tolbutamide reduced the resting conductance by 50–60%. Thus, it appears that the net K_ATP_ density is ~0.03 nS/pF with the rest representing the contribution of other ion channels (or ‘leak’ around the recording electrode). The inhibitory effect of glucose could be reversed by diazoxide and the input conductance measured in the presence of this K_ATP_ channel activator was 3- to 6-fold that measured in the presence of glucose alone in EndoC-βH1 and -βH2 cells, respectively.

The finding that high glucose and tolbutamide inhibit K_ATP_ channel activity and stimulate insulin secretion similarly should not be taken to indicate that glucose solely operates by the triggering (K_ATP_ channel-dependent) mechanism with little involvement of the amplifying (K_ATP_ channel-independent) mechanism. On the contrary, our recordings of the cytosolic [Ca^2+^]_i_ show that tolbutamide stimulates a greater number of cells than glucose does, suggesting that tolbutamide exerts a weaker stimulatory effect when normalised to the number of cells activated.

### Voltage-gated ion channels

We found that the EndoC-βH1 and -βH2 cells express voltage-gated ion channel counterparts very similar to that found in primary human beta-cells^23^. Thus, action potential firing in these human beta-cell lines involves activation of voltage-gated Na^+^, Ca^2+^ and K^+^ channels^34^. Interestingly, most of EndoC-βH1 cells exhibit large-amplitude and long-duration action potentials. Using voltage-clamp command pulses based on recorded glucose-induced action potentials, we found that this correlated with delayed and reduced activation of an iberiotoxin-sensitive K^+^ current (reflecting the opening of large-conductance Ca^2+^-activated K^+^ channels; BK) and slower action potential repolarisation. The same analysis also suggests that Kv2 channels play a relatively more prominent role in action potential repolarisation in these cell lines than in human primary beta cells^23^. Voltage-clamp analyses of the current responses further suggest that potential depolarisation principally reflects opening of voltage-gated P/Q- and L-type Ca^2+^ channels with little contribution by voltage-gated Na^+^ channels. This is because action potentials originate from fairly depolarised membrane potentials (−55 to −50 mV). At these membrane potentials, the Na^+^ channels are largely inactivated, which explains why I_Na_ contributes only marginally to action potential firing at steady-state.

### Correlating electrophysiology with gene expression

To establish how authentic EndoC-βH1 and -βH2 cells are as models of primary human beta-cells, we correlated the electrophysiological data with gene expression. We found that there was, in general, good agreement at the transcript level between both cell lines and primary human beta-cells. Among the voltage-gated Na^+^ channels, *SCN8A* (Nav1.6) and *SCN9A* (Nav1.7) are expressed at particularly high levels. We detected high expression of L-type Ca^2+^ channel α-subunits *CACNA1C* (α1C), *CACNA1D* (α1D) and P/Q-type Ca^2+^ channels *CACNA1A*. Furthermore, very high expression of the T-type Ca^2+^ channel *CACNA1H* was detected. Expression of *CACNA1E* and *CACNA1B* (encoding R- and N-type Ca^2+^-channels, respectively) were low in EndoC-βH1 and -βH2 cells (echoing what is seen in primary human beta-cells^23,33^).

Of the voltage-gated K^+^ channels, *KCNB2* (Kv2.2), *KCNQ2* (Kv7.2) and *KCNH2* (Kv11.1) are expressed at particularly high levels, mirroring what is seen in human beta-cells^33^. High expression was also observed for the BK α-subunit gene *KCNMA1*. However, despite the evidence for reduced BK activity in EndoC-βH1 cells compared to EndoC-βH2 cells, mRNA levels were comparable between the two cell lines. Overall, the expression of these voltage-gated ion channels in EndoC-βH1 and -βH2 cells mirror that seen in primary human beta-cells^32,33^.

### Correlating ultrastructure, exocytosis and insulin secretion

Using immunofluorescence as well as electron microscopy, we show that both cell lines are well granulated, although the granules are not evenly distributed within the cell. The secretory granules are found at higher density in cytoplasmic projections (Supplemental Figs 6–8). Granules are morphologically heterogeneous with a majority characterised by a very thin halo surrounding the electron dense core (insulin). The granule density (*N*_A_) can be converted to the volume density (*N*_*V*_) (Eq. 3) and corresponded to ~13 and 20 granules.μm^−3^ in EndoC-βh1 and βh2, respectively. When multiplied by the extranuclear cell volume, these values convert to ~6,000 and 15,000 secretory granules/cell in EndoC-βH1 and -βH2 cells, respectively. This is not too different from the numbers found in primary beta-cells^35,36^. Given that between 4 to 7% of the vesicles appeared to be directly in contact with the plasma membrane, we estimate the docked granule pool is of 250–650 vesicles, in fair agreement the 600 docked granules estimated in human primary beta cells^2,37^.

### Exocytosis

The measurements of exocytosis also indicate that the late steps of insulin release largely recapitulate what is observed in primary beta-cells. However, EndoC-βH1 cells could be distinguished from the EndoC-βH2 cells by the kinetics of secretion. Whereas most of exocytosis in the EndoC-βH2 cells occurred during the actual depolarisation (‘phasic exocytosis’), exocytosis in many EndoC-βH1 cells occurred after the depolarisation and when the membrane potential had returned to −70 mV (‘asynchronous exocytosis’). This suggests that the kinetics of exocytosis in EndoC-βH1 mirrors the depolarisation-evoked [Ca^2+^]_i_ transients, which typically takes a few seconds to return to basal^38^. This is consistent with the high expression of the high-affinity Ca^2+^-sensor synaptotagmin 7 (*SYT7*) in EndoC-βH1 cells. However, this cannot be the sole explanation as equally high expression of *SYT7* is seen in EndoC-βH2 cells and yet exocytosis was ‘phasic’.

Quantitatively, the exocytotic responses observed in EndoC-βH1 and -βH2 cells are impressive. A single 800 ms depolarisation produced a capacitance increase of 100 and 180 fF in EndoC-βH1 and -βH2 cells, respectively. The granule area estimated from electron microscopy was 0.12 µm^2^. With a specific capacitance of 10 fF.μm^−2^, this granule area predicts that each granule should add 1.2 fF of capacitance upon fusion. Thus, the exocytotic responses equate to the discharge of 80–150 granules, which corresponds to 15–25% of the docked vesicles pool and ~1% of the total granule number. This echoes the size of the Ready Releasable Pool (RRP, ~200 vesicles) described in human pancreatic beta cells^37^. From the relationship between pulse duration and exocytosis, we estimate that a 10 ms depolarisation (the approximate duration of an action potential) will produce an exocytotic response of ≤1% of that produce by the 800 ms pulse (Fig. 5). Thus, we can estimate that each action potential will (on average) result in the release of 0.8–1.5 granules. Multiplying these values with the observed action potential frequency (1.45 Hz) suggests that EndoC-βH1 and -βH2 cells release an astonishing 100% and 50% of their total insulin content per hour. However, the [Ca^2+^]_i_ measurements suggest that only 20–25% of the cells respond to glucose. This implies that EndoC-βH1 and -βH2 respectively release about 20% and 10% of their insulin content per hour, in fair agreement with the secretion rates actually observed.

### Impact of ‘polyhormonality’

At the mRNA level, glucagon and somatostatin levels equal that of insulin in EndoC-βH2. However, at the protein level only a relatively small fraction of cells are polyhormonal. Immunogold labelling in conjunction with electron microscopy revealed that these cells could store both insulin and somatostatin within the same granules. Interestingly, there was also relatively high expression of the somatostatin receptors 1, 2 and 3 (*SSTR1-3*), raising the possibility of autocrine inhibition. Indeed, when the cells were treated with 1 μmol/L CYN154806, glucose-induced insulin secretion was doubled. As this antagonist is selective for SSTR2, it is possible that even greater enhancement would have been observed if SSTR1 and SSTR3 had also been targeted.

## Conclusion

We conclude that both EndoC-βH1 and -βH2 cells are reliable models of primary human beta-cells. We acknowledge that the secretory response to glucose and tolbutamide appears relatively limited and subjected to batch-to-batch variations. This is illustrated by the variable GSIS fold stimulation at high glucose reported in different studies^8,21,39^ which could even approach that seen in primary beta-cells^16^. Electrophysiologically, both EndoC-βH1 and -βH2 cells closely mirror primary human beta-cells both at the functional and the gene expression levels. Our detailed characterisation of both cell lines demonstrates that they represent versatile models of primary human beta-cells which can be used for high-throughput pipelines^18,21^ as well as detailed *in vitro* studies of the insulin secretory defects^19,40^. The fact that they are relatively easy to modify genetically makes them an attractive and powerful tool for future investigations of the relationship between gene expression and beta-cell function.

## Electronic supplementary material

Supplementary Material

Supplementary Dataset

## Data Availability

The transcriptomic datasets generated during and analysed during the current study are available in the at the European Nucleotide Archive (https://www.ebi.ac.uk/ena) under the accession number PRJEB23293 (a summary of the transcriptome analysis can also be found in Supplementary dataset).
