# Supplementary material for: Electrophysiological properties of human beta-cell lines EndoC-βH1 and -βH2 conform with human beta-cells
Source: Sci Rep. 2018 Nov 19;8:16994. doi: 10.1038/s41598-018-34743-7 (PMC6242937; doi:10.1038/s41598-018-34743-7)
Supplement: Supplementary file 1 — Supplementary Material [file 41598_2018_34743_MOESM1_ESM.docx]

**Supplementary Material**

**Electrophysiological properties of human beta-cell lines EndoC-βH1 and ‑βH2 conform with human beta-cells**

Benoît Hastoy*, Mahdieh Godazgar, Anne Clark, Vibe Nylander, Ioannis Spiliotis, Martijn van de Bunt, Margarita Chibalina, Amy Barrett, Carla Burrows, Andrei Tarasov, Raphael Scharfmann, Anna L. Gloyn, Patrik Rorsman.

*Corresponding author: Benoît Hastoy: [benoit.hastoy@ocdem.ox.ac.uk](mailto:benoit.hastoy@ocdem.ox.ac.uk), [tel: 0044](tel:0044) 1865 857256

**Supplementary Figures:**

Supplementary Figure S1 – Expression of genes involved in glucose sensing.

Supplementary Figure S2 – Expression of genes encoding voltage-gated Na^+^ channel subunits

Supplementary Figure S3 – Expression of genes encoding voltage-gated Ca^2+^ channel subunits.

Supplementary Figure S4 – Expression of genes encoding subunits of K^+^ channels.

Supplementary Figure S5 – Expression of proteins-involved in exocytosis.

Supplementary Figure S6 – Polyhormonality of EndoC-βH1 and -βH2 cells

Supplementary Figure S7 – Hormone and membrane receptor expression in EndoC-βH1 and -βH2 cells.

Supplementary Figure S8 – Electron micrograph of EndoC-βH1 cultured on filter.

Supplementary Figure S9 – Expressions of foetal genes in EndoC-βH1, -βH2 and publically available human foetal and adult datasets.

Supplementary Dataset – EndoC-βH1 and -βH2 RNA sequencing datasets.

**Supplementary methods:**

- Transfections
- [Ca^2+^] imaging
- RNA sequencing
- Electron microscopy

**Supplementary Figures**

**
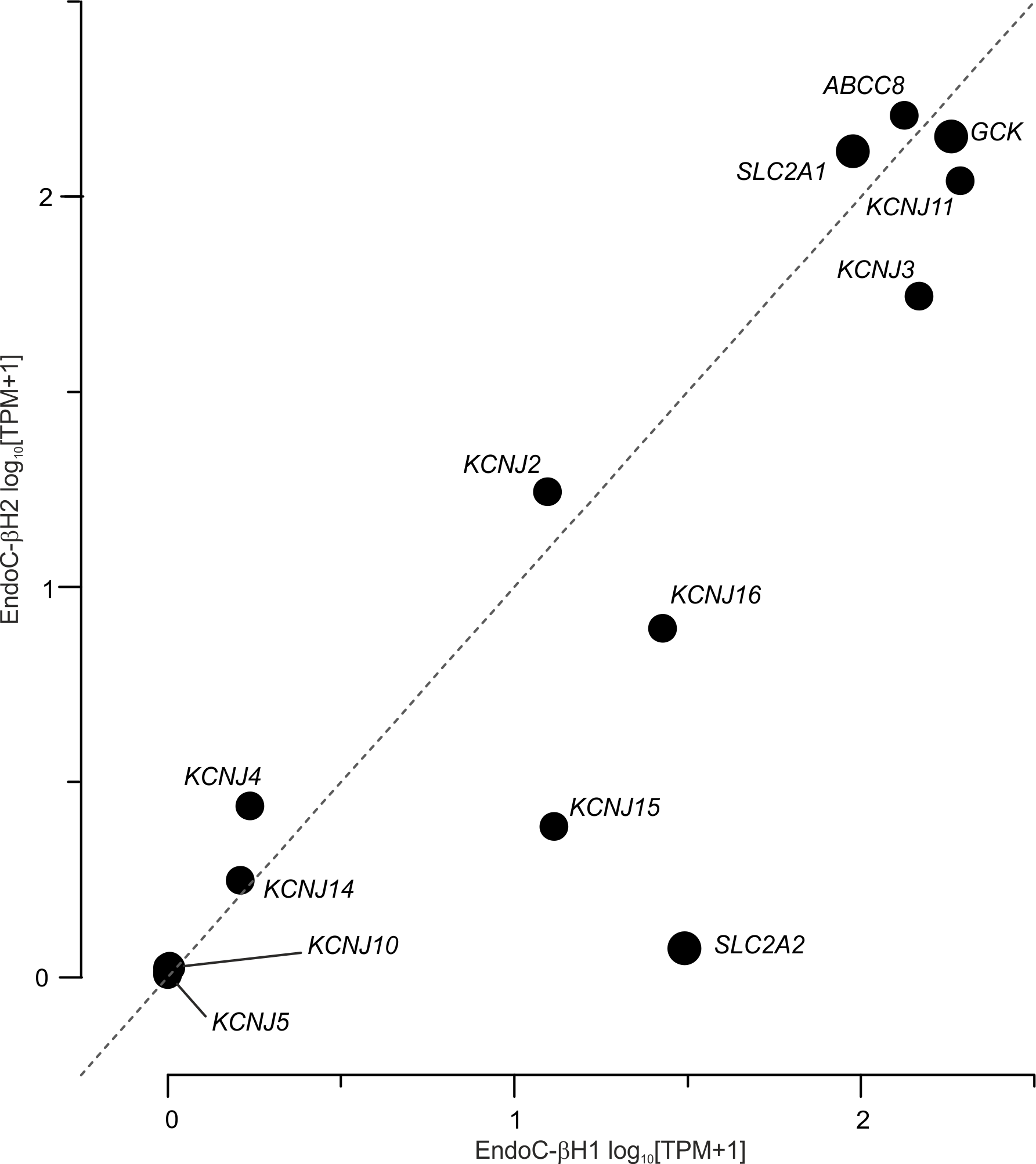
Supplementary Figure S1** – Expression of genes involved in glucose sensing. GCK, glucokinase; SLC2A1, GLUT1; SLC2A2, GLUT2; KCNJ3, GIRK3; KCNJ11, Kir6.2; ABCC8, SUR1. RNA-seq TPM (Transcript Per Million) values were plotted as Log_10_(TPM+1) to avoid negative values. X and Y axes correspond to EndoC-βH1 and EndoC-βH2 respectively. Dashed line represent equal expression in both cell lines.

**
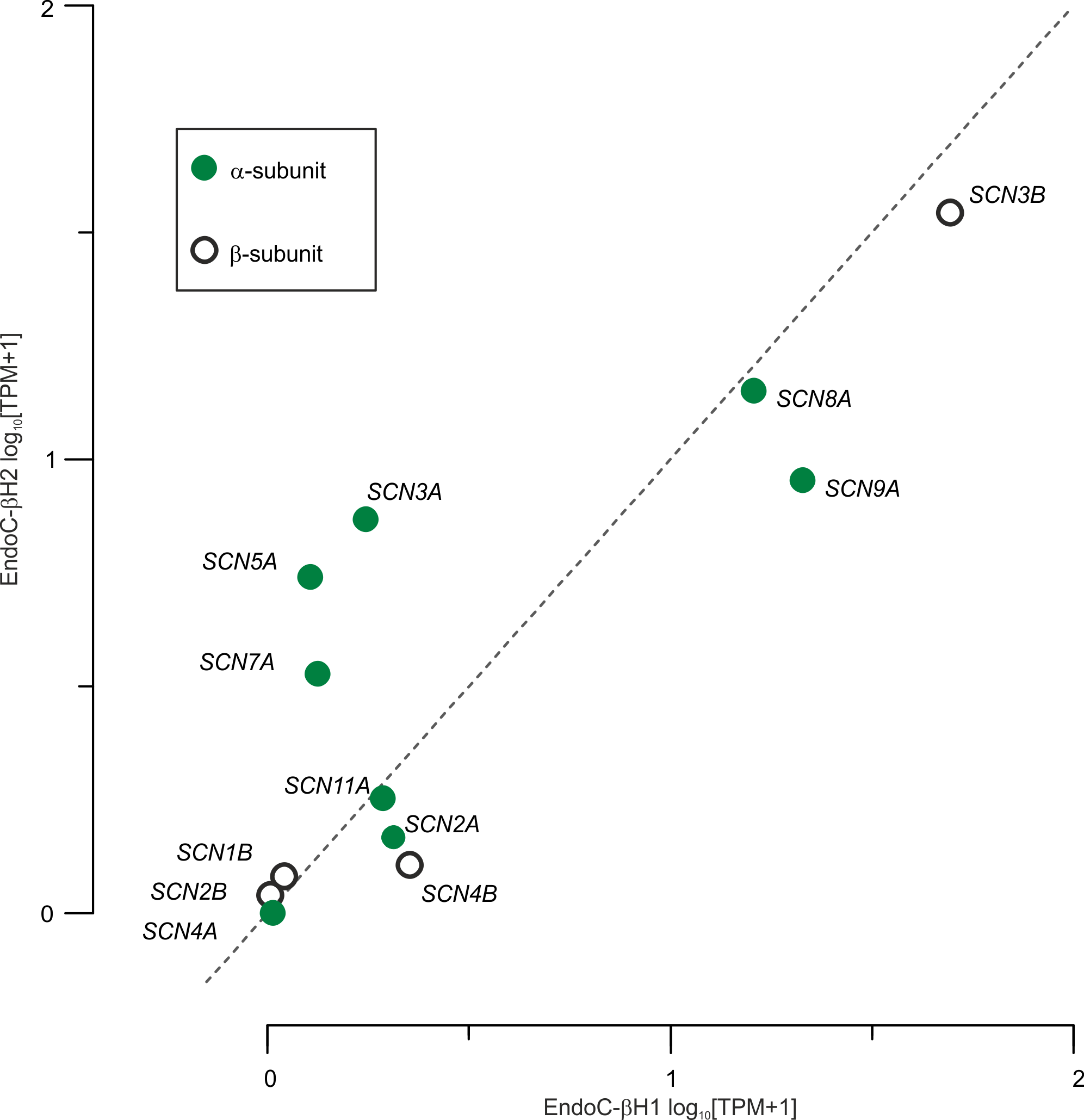
Supplementary Figure S2** – Expression of genes encoding voltage-gated Na^+^ channel subunits. SCNxA are α-subunits (green filled circles) and SCNxB are β-subunits (open circles). Data expressed as in Supplementary Figure S1.

**
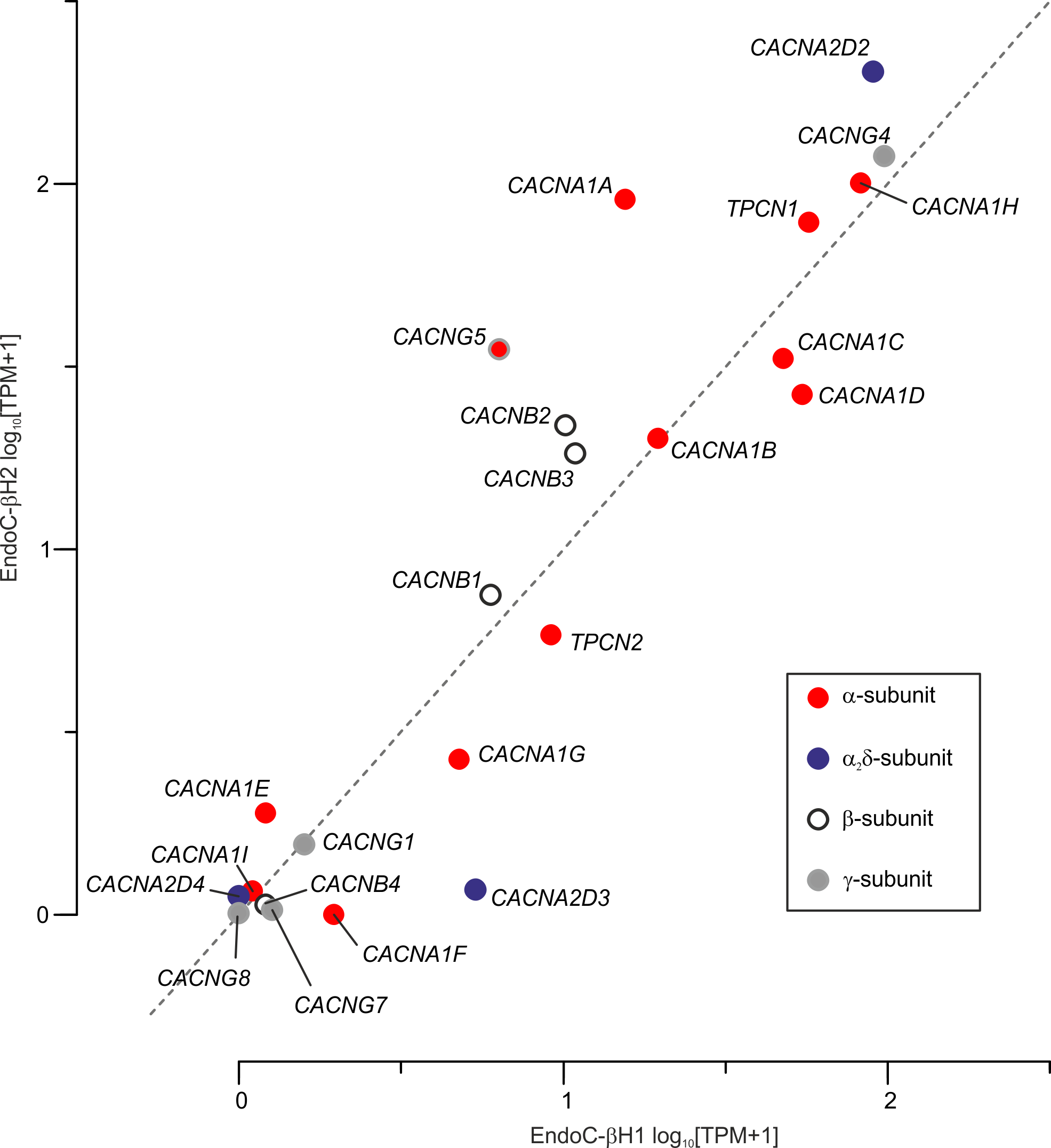
Supplementary Figure S3** – Expression of genes encoding voltage-gated Ca^2+^ channel subunits. Expression of pore-forming α_1_-subunits (CACNA1x, red filled circles). Expression of auxiliary subunits: *CACNB*x, β-subunits (open circles); *CACNG*x, γ-subunits (grey filled circles); *CACNA2D*x, α_2_δ-subunits (blue filled circles). Data expressed as in Supplementary Figure S1.

**
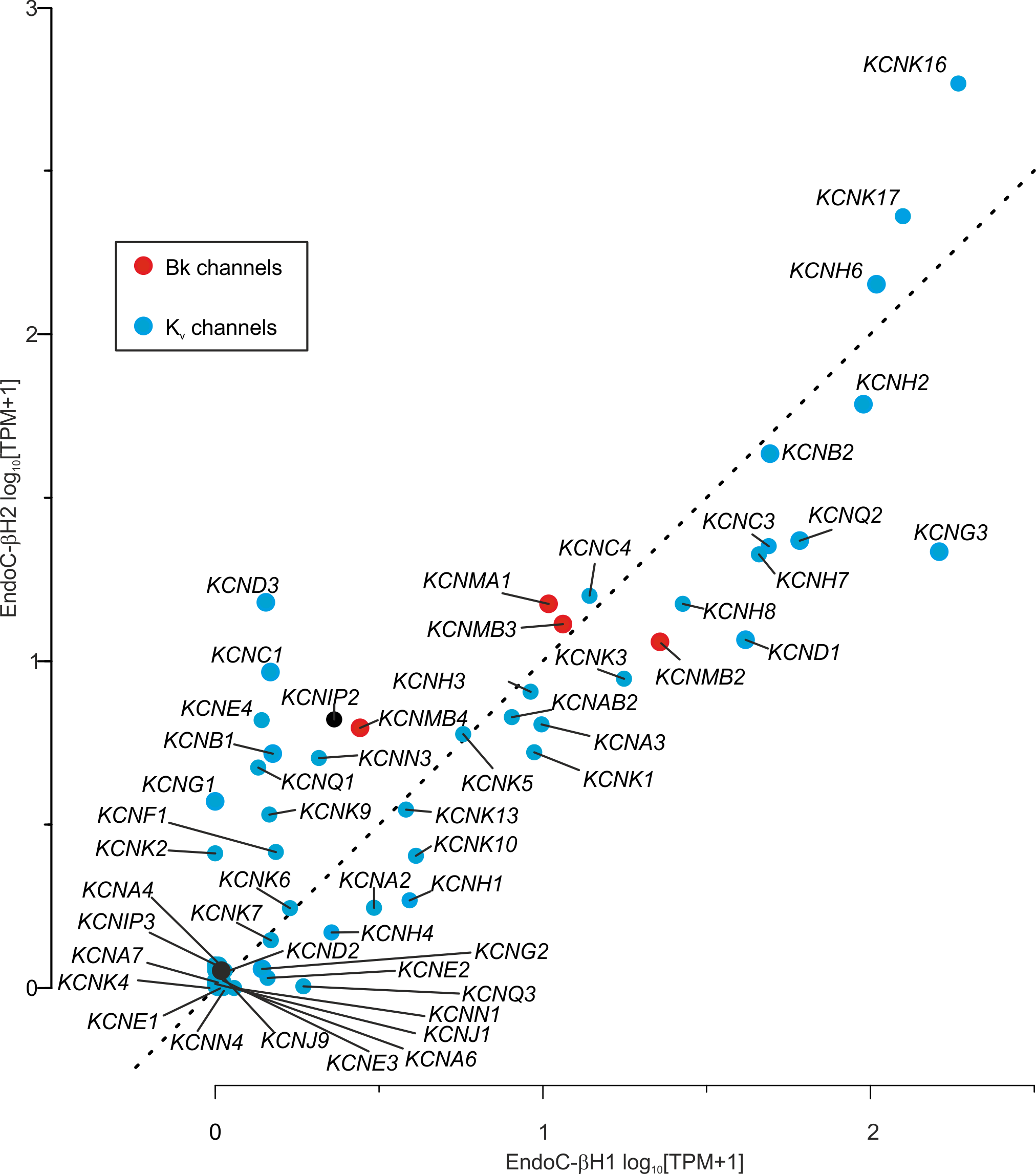
Supplementary Figure S4** – Expression of genes encoding subunits of the large-conductance Ca^2+^-activated K^+^ channels (BK; red filled circles), the pore-forming subunits of voltage-gated K^+^ channels (blue filled circles) [*KCNA*x, Kv1.x; *KCNB*x, Kv2.x; *KCNC*x, Kv3.x; *KCND*x, Kv4.x; *KCNH*x, Kv11.x (hERG); *KCNK*x, *K2Px* (2-pore channels)], and the voltage-gated K^+^ (Kv) channel-interacting proteins (KCNIP, black filled circles). Data expressed as in Supplementary Figure S1.

**
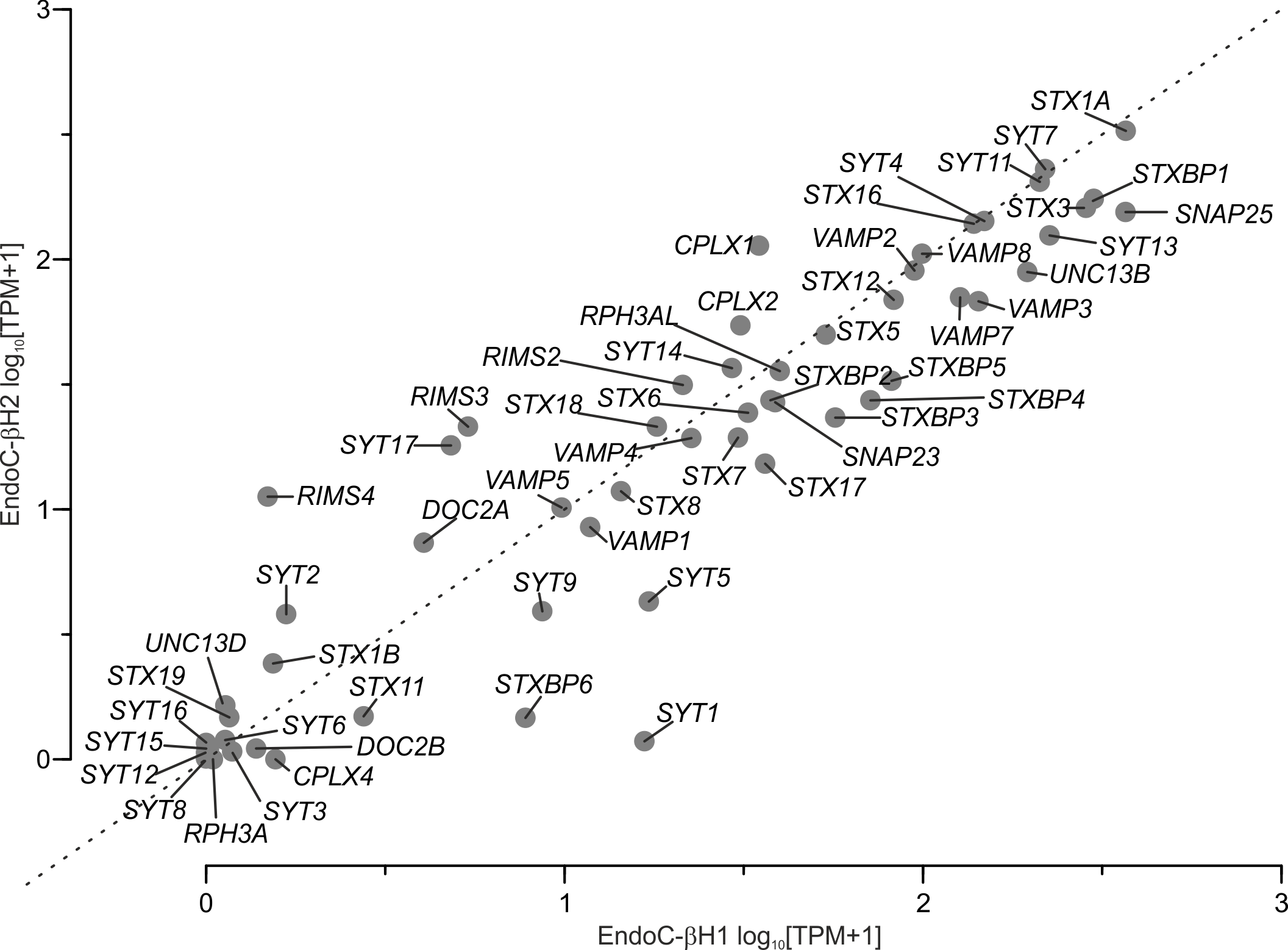
Supplementary Figure S5** – Expression of proteins involved in exocytosis. *STX*x, syntaxins; *VAMP*x, VAMPs; *CPLX*x, complexins; *STXBP*x (syntaxin-binding proteins); *SNAP*x, synaptosomal-associated protein (e.g. Munc18); *SYT*x, synaptotagmins; *UNC13*x, Munc13; *RIMS*x, RIMx; *RPH3AL*, NOC2. Data expressed as in Supplementary Figure S1.

**
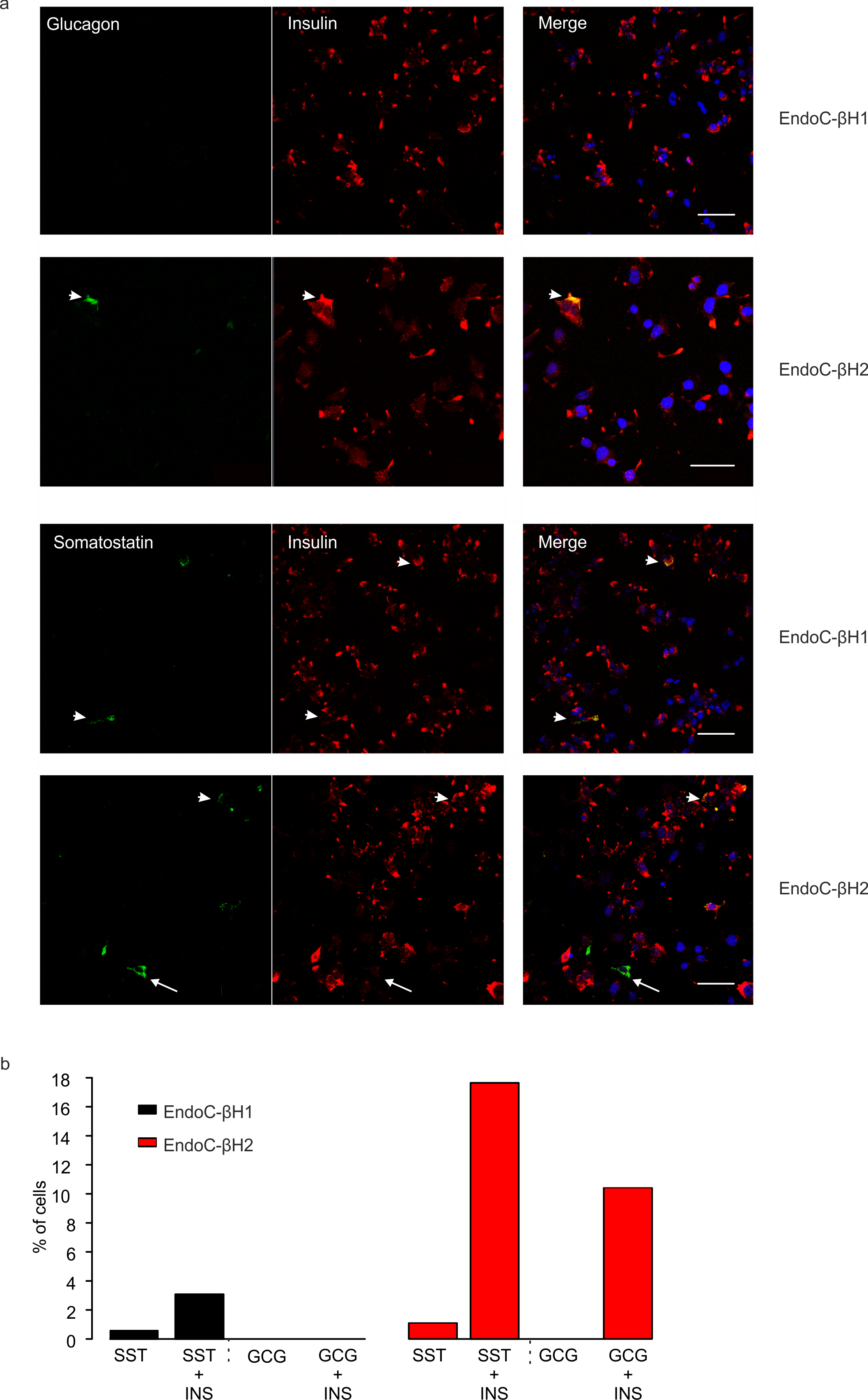
**

**Supplementary Figure S6** – Polyhormonality of EndoC-βH1 and –βH2 cells. (a). Immunofluorescent labeling of insulin (red), glucagon or somatostatin (green) in EndoC-βH1 and –βH2 cells (arrows). Scale bar 50µm. DAPI (blue labeling for nuclei). (b) Proportions of solely positive somatostatin (SST) or glucagon (GCG) cells and proportions of polyhormonal (co-localizing with insulin, INS) EndoC-βH1 and –βH2 cells (SST, n=876 and 272 cells; GCG, n=279 and 240 cells).


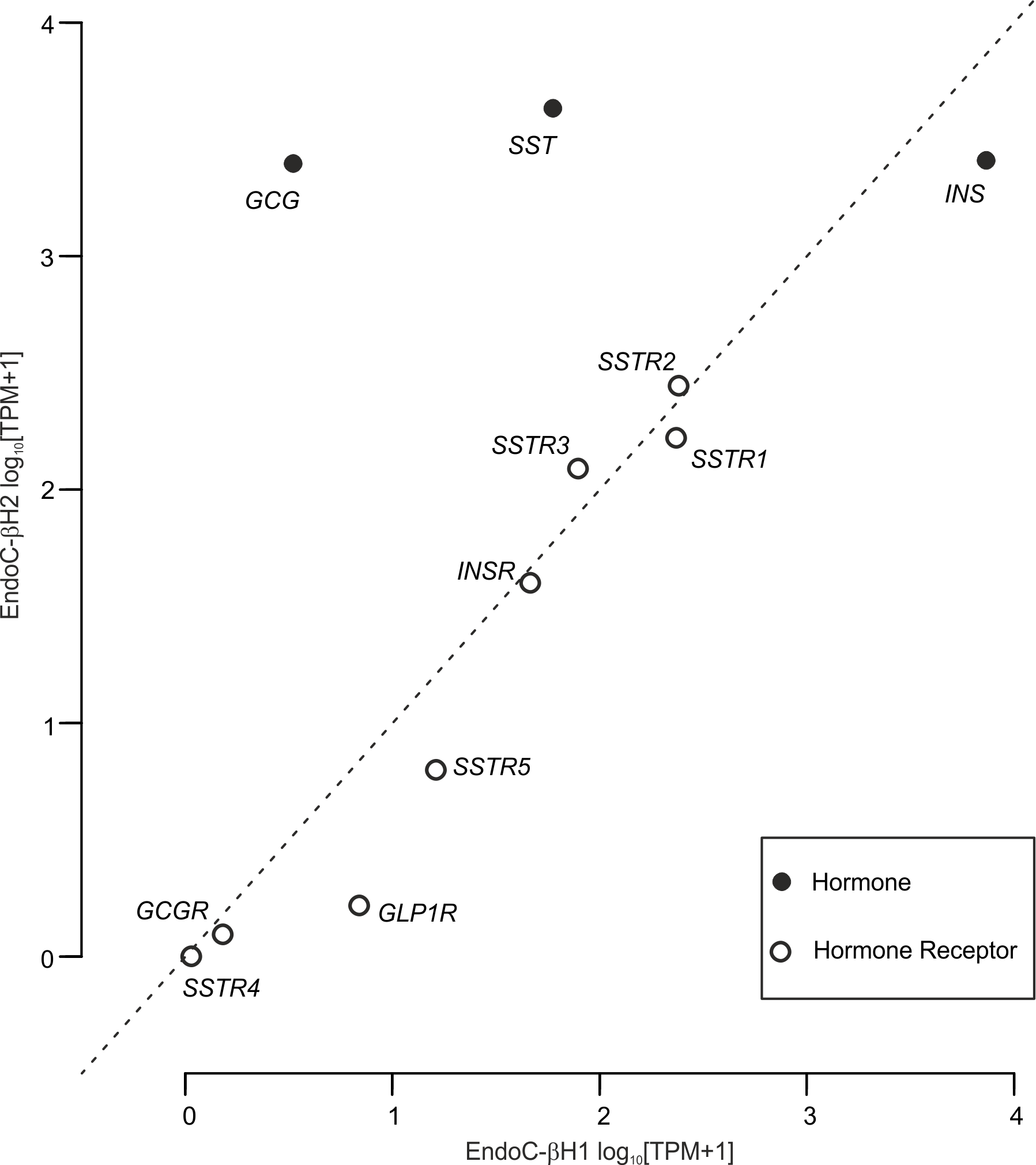


**Supplementary Figure S7** – Hormone and membrane receptor expression in EndoC-βH1 and –βH2 cells. *INS*, insulin; *GCG*, glucagon; SST, somatostatin; *INSR*, insulin receptor; *SSTR*x, somatostatin receptor; *GLP1R*, GLP-1 receptor; *GCGR*, glucagon receptor. Data expressed as in Supplementary Figure S1.


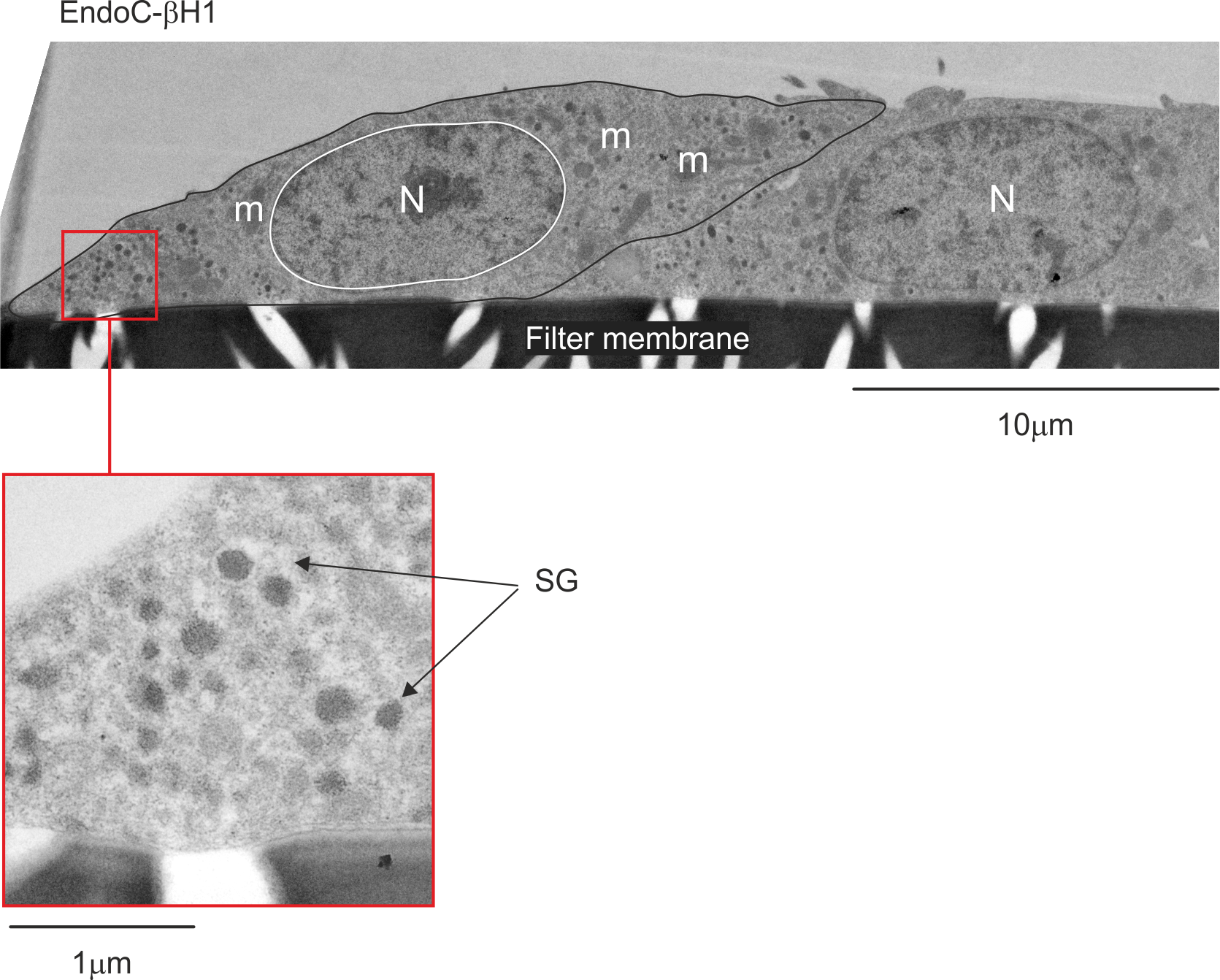


**Supplementary Figure S8** – Electron micrograph of EndoC-βH1 cultured on filter. Abbreviation: m: mitochondria, SG: Secretory Granule, N: Nucleus.


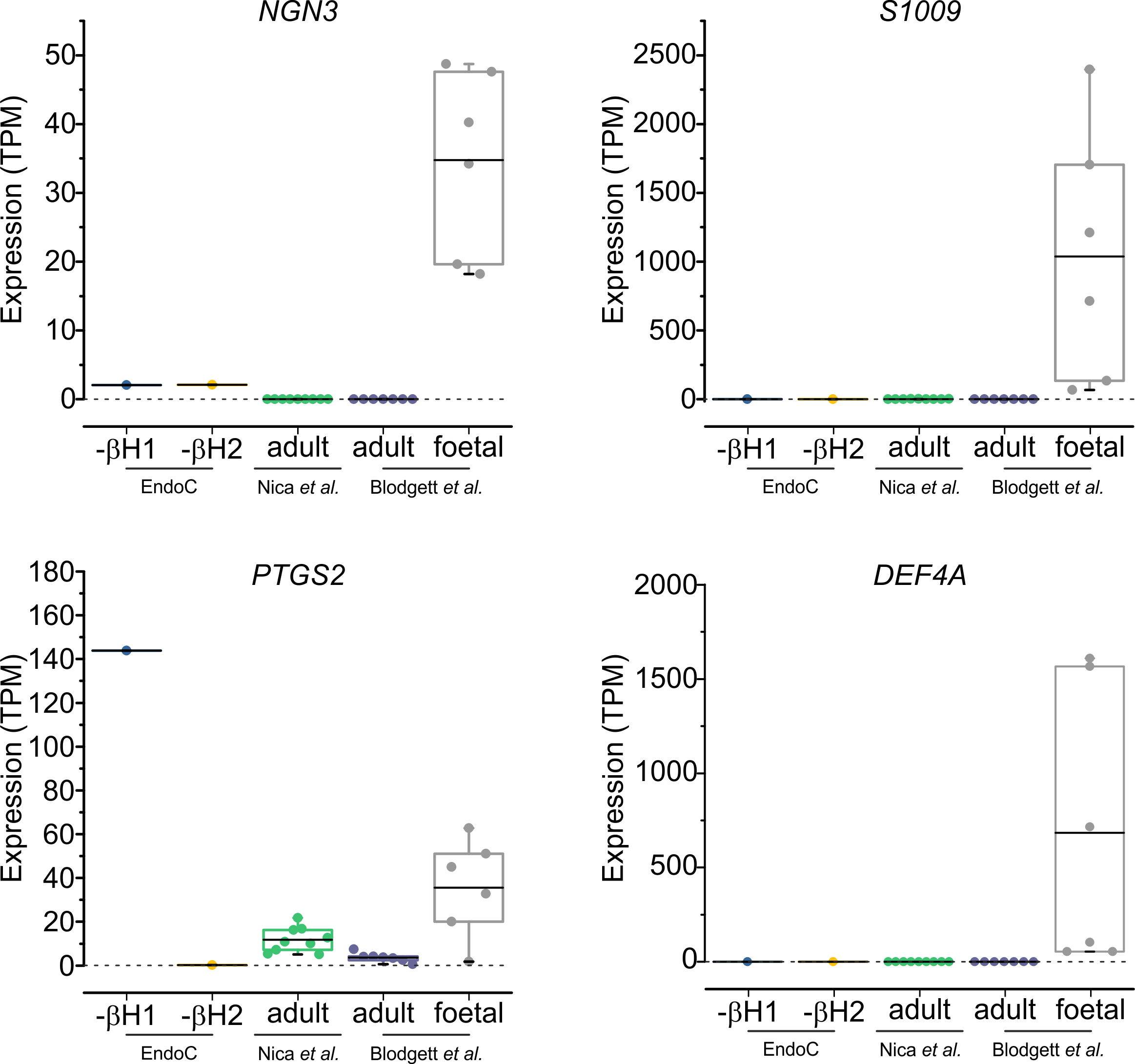


**Supplementary Figure S9** – Expression of foetal genes in EndoC-βH1, -βH2 and publically available human foetal and adult datasets. The subset of genes (*NGN3*, *S1009*, *PTGS2*, *DEF4A*) has been identified from the foetal transcriptomic dataset (Blodgett *et al.*) and has been subsequently screened against additional human adult dataset (Nica *et al.*).

**Supplementary Dataset** – EndoC-βH1 and -βH2 RNA sequencing datasets. TPM values are given for each transcript in both cell lines.

**Supplementary methods:**

**Cell lines and cell culture**

EndoC-βH1 and -βH2 cell lines, both generated from human fetal pancreatic buds were provided by Endocell and Raphael Scharfmann. Both cell lines were regularly tested for mycoplasma contamination and cultured at 5.6mmol/L glucose in Dulbecco’s modified Eagle’s medium (DMEM; Sigma-Aldrich) with 2% BSA fraction V fatty acid free (Roche Diagnostics), 50μmol/L 2-mercaptoethanol, 10mmol/L nicotinamide (Calbiochem), 5.5μg/ml transferrin (Sigma-Aldrich), 6.7ng/ml selenite (Sigma-Aldrich), 100U/ml penicillin, and 100μg/ml streptomycin. A density of 70000-75000 cells/cm² was plated on Matrigel (1%), fibronectin (2μg/ml; Sigma-Aldrich) -coated flask and cultured at 37°C and 5% CO2 for a week.

**Transfections**

For each coverslip, 25% of a mixture containing 1.25ng/μl DNA, 1% of lipofectamine diluted in 100μl Opti-MEM was gently spread in the drop of plated cells and cultured in humid chamber for 48h at 37◦C.

**[Ca^2+^] imaging**

Image sequences were analysed (cell detection, background subtraction, ROI intensity v time analysis) using opensource FIJI software. IgorPro package (Wavemetrics) was used to analysed the data and to generate the heatmaps. Fluorescence (F) was normalized to that at the start of the recording (F0) and annotated as F/F0. For quantification, the Area Under the Curve (AUC) was calculated per unit of time and used to characterize the effect of each experimental condition.

**RNA sequencing**

After poly-A selection, strand-specific libraries were prepared using the Illumina TruSeq Stranded mRNA Library Prep Kit. Libraries were sequenced to a total depth of ~50 million read pairs as a multiplex with other samples over two lanes of Illumina HiSeq2000 as 100-nucleotide paired-end reads using the Illumina TruSeq PE Cluster Generation and TruSeq SBS v3 kits. STAR version 2.5.1b 25 on was used to align sequencing reads to the human genome reference GRCh37.p13, and using GENCODE release 19 as the transcriptome reference. Gene counts were quantified using featureCounts v1.5.0-p2, and converted into TPM (Transcripts Per kilobase Million).

**Electron microscopy**

In analysed sections, as these cell lines are very polarised, cells devoid of visible granule were not included in the analysis and our value for granule density therefore represent an upper estimate. Quantification of immunogold labelling per vesicle, cross-sectional vesicle area, vesicle density NA (number of vesicles/cytoplasmic area) were determined using Image J software.
